# Optical imaging spectroscopy for rapid, primary screening of SARS-CoV-2: a proof of concept

**DOI:** 10.1038/s41598-022-06393-3

**Published:** 2022-02-18

**Authors:** Emilio Gomez-Gonzalez, Alejandro Barriga-Rivera, Beatriz Fernandez-Muñoz, Jose Manuel Navas-Garcia, Isabel Fernandez-Lizaranzu, Francisco Javier Munoz-Gonzalez, Ruben Parrilla-Giraldez, Desiree Requena-Lancharro, Pedro Gil-Gamboa, Cristina Rosell-Valle, Carmen Gomez-Gonzalez, Maria Jose Mayorga-Buiza, Maria Martin-Lopez, Olga Muñoz, Juan Carlos Gomez-Martin, Maria Isabel Relimpio-Lopez, Jesus Aceituno-Castro, Manuel A. Perales-Esteve, Antonio Puppo-Moreno, Francisco Jose Garcia-Cozar, Lucia Olvera-Collantes, Raquel Gomez-Diaz, Silvia de los Santos-Trigo, Monserrat Huguet-Carrasco, Manuel Rey, Emilia Gomez, Rosario Sanchez-Pernaute, Javier Padillo-Ruiz, Javier Marquez-Rivas

**Affiliations:** 1 Department of Applied Physics III, ETSI School of Engineering, Universidad de Sevilla, Camino de los Descubrimientos s/n, 41092 Sevilla, Spain; 2 Institute of Biomedicine of Seville (IBIS), 41013 Sevilla, Spain; 3 School of Biomedical Engineering, The University of Sydney, Sydney, NSW 2006 Australia; 4 Unidad de Producción y Reprogramación Celular (UPRC), Red Andaluza de Diseño y Traslación de Terapias Avanzadas, Consejería de Salud y Familias, Junta de Andalucía, 41092 Sevilla, Spain; 5 EOD-CBRN Group, Spanish National Police, 41011 Sevilla, Spain; 6 Technology and Innovation Centre, Universidad de Sevilla, 41012 Sevilla, Spain; 7 Service of Intensive Care, University Hospital ‘Virgen del Rocio’, 41013 Sevilla, Spain; 8 Department of Medicine, College of Medicine, Universidad de Sevilla, 41009 Seville, Spain; 9 Service of Anesthesiology, University Hospital ‘Virgen del Rocio’, 41013 Sevilla, Spain; 10 Department of Surgery, College of Medicine, Universidad de Sevilla, 41009 Seville, Spain; 11 Instituto de Astrofísica de Andalucía, CSIC, 18008 Granada, Spain; 12 Department of Ophthalmology, University Hospital ‘Virgen Macarena’, 41009 Sevilla, Spain; 13 OftaRed, Institute of Health ‘Carlos III’, 28029 Madrid, Spain; 14 Centro Astronomico Hispano Alemán, 04550 Almeria, Spain; 15 Department of Electronic Engineering, ETSI School of Engineering, Universidad de Sevilla, 41092 Sevilla, Spain; 16 Department of Biomedicine, Biotechnology and Public Health, University of Cadiz, 11003 Cadiz, Spain; 17 Instituto de Investigación e Innovación Biomedica de Cádiz (INIBICA), 11009 Cadiz, Spain; 18 Corporación Tecnológica de Andalucía, 41092 Sevilla, Spain; 19 CER ‘Dr. Gregorio Medina Blanco’, 41807 Espartinas, Sevilla Spain; 20 CAMBRICO BIOTECH, 41015 Sevilla, Spain; 21 Joint Research Centre, European Commission, 41092 Sevilla, Spain; 22 Department of General Surgery, University Hospital ‘Virgen del Rocío’, 41013 Sevilla, Spain; 23 Service of Neurosurgery, University Hospital ‘Virgen del Rocío’, 41013 Sevilla, Spain; 24 Centre for Advanced Neurology, 41013 Sevilla, Spain

**Keywords:** Viral infection, Near-infrared spectroscopy, Near-infrared spectroscopy

## Abstract

Effective testing is essential to control the coronavirus disease 2019 (COVID-19) transmission. Here we report a-proof-of-concept study on hyperspectral image analysis in the visible and near-infrared range for primary screening at the point-of-care of SARS-CoV-2. We apply spectral feature descriptors, partial least square-discriminant analysis, and artificial intelligence to extract information from optical diffuse reflectance measurements from 5 µL fluid samples at pixel, droplet, and patient levels. We discern preparations of engineered lentiviral particles pseudotyped with the spike protein of the SARS-CoV-2 from those with the G protein of the vesicular stomatitis virus in saline solution and artificial saliva. We report a quantitative analysis of 72 samples of nasopharyngeal exudate in a range of SARS-CoV-2 viral loads, and a descriptive study of another 32 fresh human saliva samples. Sensitivity for classification of exudates was 100% with peak specificity of 87.5% for discernment from PCR-negative but symptomatic cases. Proposed technology is reagent-free, fast, and scalable, and could substantially reduce the number of molecular tests currently required for COVID-19 mass screening strategies even in resource-limited settings.

## Introduction

The widespread community transmission of the coronavirus disease (COVID-19) has forced nations around the globe to impose severe measures including lockdown periods^1^, border closures^2^, and mass screening^3^ to name a few. The vast geographical spread of the severe acute respiratory syndrome coronavirus 2 (SARS-CoV-2) and the large number of asymptomatic carriers are among the causes hampering the eradication of the disease. Fortunately, identification and isolation of confirmed cases has demonstrated efficacy in controlling local outbreaks while reducing its propagation^4^ until the achievement of herd immunity allows a return to normality^5^.

In an unprecedented scenario in our lifetime, the COVID-19 pandemic has stimulated biomedical research to find technological solutions to manage such a global threat. The development of reliable, fast and scalable detection tools is among the top priorities in fighting the spread of the virus, as an increased screening and identification capacity becomes critical in containing outbreaks^6^, and effective and extended testing procedures are required to improve epidemiological models^7^ and support public health decisions.

### Screening and diagnostic tests

From the perspective of disease management, two main categories of examinations are usually considered, namely screening and diagnostic tests^8^. Screening exams are intended to detect early disease or asymptomatic carriers, i.e., to identify individuals who have the target disease, but which may not have any sign or symptom of it. In a viral infection scenario, the goal of screening tests is the identification of infected individuals to avoid that they may propagate the disease, and they are usually given to asymptomatic individuals^9^ or to those without known exposure to the pathogen^10^. To be effective, screening tests should detect infectious subjects before the onset of symptoms and be as broadly applied as possible within the population under analysis. They should not be invasive preferably and be easy to use. Screening tests document ‘an estimate of the level of risk and determine whether a diagnostic test is justified’^11^. False positives are considered acceptable for high-sensitivity screening tests, ‘particularly if they are not harmful nor expensive’^11^. Diagnostic tests are intended to diagnose the target disease or condition on the evaluated individual, so that proper clinical measures can be taken. They are required to provide diagnostic precision and accuracy, even at high cost and discomfort for patients. They are also applied after positive results from a screening test^8^. In a viral infection scenario, diagnostic tests are performed when there are reasons to suspect that an individual may be infected, for example with symptoms, or having been exposed to the pathogen (e.g., close contacts of confirmed cases), in a high-risk group (e.g., health care personnel), or to determine the stage and evolution of the infection in a sick subject.

From an operational perspective, diagnostic tests for COVID-19 can be grouped in three major categories: molecular tests (based on detection of the genetic material of the virus, mainly performed on nasal and throat swabs), antigen tests (which detect specific proteins of the SARS-CoV-2 virus, also performed on throat swabs), and serology tests (immunoassays on blood samples that detect certain specific viral antibodies, quantify the immune response and assess the level of immunity).

The leading diagnostic test is laboratory-based polymerase chain reaction (PCR), which is a nucleic acid amplification test, i.e., a molecular exam, that has become the common ‘gold standard’ for the detection of SARS-CoV-2, providing excellent sensitivity and specificity while relatively inexpensive. However, it requires complex equipment and reagents, and it is usually performed by highly skilled personnel at centralized laboratory facilities which receive submitted samples, with turnaround time for results from several hours to days. There are also some test kits for use at the point-of care or at-home which provide results in less than an hour, but reagents require careful storage and handling.

To overcome some of the drawbacks of molecular tests, hundreds of rapid protein-based diagnostic devices have emerged^12^. Among them, enzyme-linked immunosorbent assays or lateral flow assays can detect SARS-CoV-2 specific human antibodies or viral antigens^13^. They are also available for at-home use and provide results in 15 min, but their performance is substantially poorer, with lower sensitivity than PCR for the detection of mild cases and significant false negatives. Negative antigen tests are usually followed by molecular (e.g., PCR) or repetitive checks along several days for confirmation^14^.

More sophisticated approaches integrate molecular technologies that range from clustered regularly interspaced short palindromic repeats (CRISPR)-based strategies^15,16^ to improve nucleic acid detection^17^, to the use of functionalized nanomaterials^18,19^. However, they are not used for mass screening. Recent innovative approaches for COVID-19 diagnostic also include the combination of health data provided by wearable sensors and self-assessment of symptoms^20^.

### Optical technologies for viral detection

Optical spectroscopic methods have been developed to detect viruses in vegetal structures^21–23^ and in human samples, mostly in blood. They involve polarimetric and fluorescence spectroscopy, and different implementations of Raman spectroscopy for identification of Dengue virus^24^, hepatitis B and C viruses^25^, and microfluidic devices to recognize avian influenza A and other respiratory infections^26^. Further approaches propose the use of nanomaterials targeted to specific viral antibodies to enhance the potentialities of optical spectroscopy to detect the human immunodeficiency virus^27^. Simpler experimental set-ups pose great interest to develop easy-to-implement testing units suitable for use at the point-of-care, and diffuse reflectance spectroscopy has been applied to discern mosquitos fed with human blood containing Zika virus^28^ from controls, although signals from potential alterations in tissues and structures due to the infection remain to be discerned from those arising from the virus itself.

Since the current pandemic emerged, many innovative optical and photonic techniques are being specifically developed for detection of SARS-CoV-2. Complex microscopy imaging setups have been described to explore Raman scattering measurements, fluorescence imaging and surface plasmon resonance, Fourier-transform infrared spectroscopy and colorimetry^29–32^. Remarkably significant results for fast COVID-19 diagnosis have been obtained using attenuated total reflection Fourier transform infrared spectroscopy of RNA extracts from nasopharyngeal samples combined with machine learning data analysis^33^, and of pharyngeal saliva samples processed with genetic algorithm-linear discriminant analysis^34^. Other optical-related detection schemes rely on nanomaterials-enhanced sensing within lab-on-chip devices^29^ and combined with hyperspectral microscopy imaging^35^, matrix-assisted laser desorption/ionization time-of-flight mass spectrometry^36^ or employ fiber optics probes to record diffuse reflectance spectra^28^. A previous work^37^ by the same authors showed that hyperspectral imaging of diffuse reflectance in the visible and near-infrared ranges can be used to detect a synthetic viral model commonly employed for the study of SARS-CoV-2.

Advances in the knowledge of the mechanisms of airborne spread of the disease have led to research on rapid methods for detecting the SARS-CoV-2 virus and indirect related markers in exhaled air and salivary aerosols. Aimed at developing potential non-contact, fast breath analyzers (‘breathalyzers’), they rely on many different technologies, from well-established gas chromatography, mass spectrometry and photonics biosensors to new concepts of ‘electronic nose’ sensing and terahertz spectroscopy^38^.

### Unmet needs in the COVID-19 pandemic

While there are available diagnostic tests to determine if an individual has an active COVID-19 infection, worldwide spread of the virus and its variants—outpacing most public health measures—shows that there remains an urgent need of easily deployable screening tests to perform mass, repetitive (time seriated) checks to detect asymptomatic infectious individuals. Of particular importance is the identification of those subjects at initial stages—before the onset of symptoms—and the so-called ‘super-carriers’, asymptomatic individuals with a very high viral load, potential ‘super-spreaders’ of the disease^39^.

From national to local and community levels, many types of COVID-19 screening programs are being implemented, even using tests authorized under emergency approvals (or not approved), with varying levels of success. Criteria for this type of extensive testing include high sensitivity and rapid turnaround time, and the (sometimes difficult) availability of authorized molecular tests for confirmation of positive and ‘concerning negative’ cases^40^.

In addition, virus mutations generate different strains which may modify its ability to spread, the severity of related diseases and the performance of public health measures. Unfortunately, by December 2021 there are not any tests authorized (by the United States Food and Drug Administration) to detect specific SARS-CoV-2 variants^14^, not even for those categorized by the World Health Organization as ‘variants of concern’ (Alpha, Beta, Gamma, Delta, and Omicron) of the disease^41^. The current COVID-19 pandemic has underlined the significance of searching for easy-to-implement testing tools potentially useful for real-life applications, particularly at the point-of-care and in constrained resource settings.

### An approach based on light scattering

In the present study, we present a proof of concept of the use of optical diffuse reflectance hyperspectral imaging in the visible and near infrared ranges combined with specific data analysis as a new technique for fast primary screening of SARS-CoV-2 in 5-µL fluid samples deposited on a surface. This approach builds upon (i) the expanding area of hyperspectral imaging^42^ and their different experimental set-ups and processing approaches^43^, particularly on close-range^44,45^ reflectance applications^46^, (ii) advances on light scattering techniques for the analysis of size and structure of elements (e.g., bacteria, viruses) below the wavelength of the employed light^47^, and (iii) the aforementioned previous work by the same authors^37^ that demonstrated how the same methodology can be exploited for detection and quantification of a SARS-CoV-2 model in two biofluids (phosphate buffered saline solution and artificial saliva), both as liquid droplets and dry residues.

Optical diffuse reflectance results from the complex phenomena of interaction resulting from light incident on thin fluid samples. The mainly involve reflection, refraction, elastic and inelastic (Raman) scattering, absorption, and re-emission (fluorescence), strongly modulated by specific features of the samples, i.e., by the presence of elements with varying degrees of sizes, shapes and potential physical, biological and biochemical crossed interactions in in the solution^48^. In the present study we surveyed samples of water-based, optically transparent biofluids with viruses (namely, synthetic viral models in saline solution and in artificial saliva, and samples of SARS-CoV-2-positive human nasopharyngeal exudate and fresh saliva, and their corresponding negative controls). Samples were all deposited as relatively small (5 uL) liquid droplets on a supporting plate, and it was hypothesized that most useful information would arise from sub-surface scattering, a physical process of much interest for computer-graphics rendering, usually modeled using the radiative transfer framework^49^. Note that the diameter of viral particles (and its engineered models) is about 120–140 nm, below the lowest wavelength of the employed illumination (< 400 nm) and, therefore, optical imaging of individual particles is not feasible and its visualization would require electron microscopy.

We have explored hyperspectral image analysis in the visible and near-infrared (VNIR) band of the electromagnetic spectrum because it requires relatively simple optical imaging technology, potentially useful for deployment of easy-to-implement point-of-care devices. However, in the VNIR band, differences among reflectance spectra from positive samples and their negative controls are difficult to discern following the standard spectroscopy approach, that is, looking for distinctive features (i.e., peaks or absorption bands). Instead, we have analyzed averaged differences of pixel spectra relative to the background, integrating them to droplet and patient levels. Using this procedure, conceptually similar to the per-layer information extraction from the multiple, noisy signals from neurons in brain-computer interfaces^50,51^, it was possible to effectively enhance the embedded information that allowed detecting the presence of the virus and its quantification^37^.

## Results

**Figure 1:**
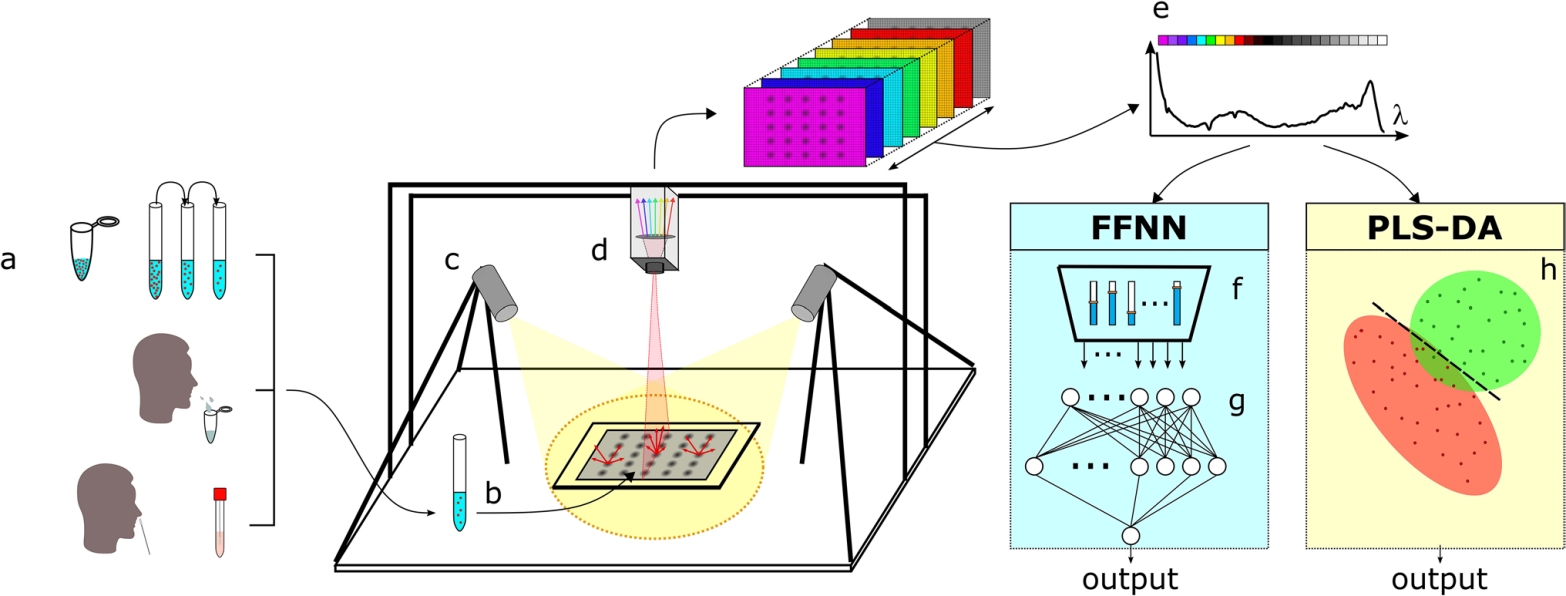
Schematic of the hyperspectral imaging assay. (**a**) Three different types of samples were analyzed. Top, samples containing spike pseudotyped lentiviral particles (i.e., synthetic coronaviruses). Middle, human saliva of SARS-CoV-2 suspects. Bottom, inactivated nasopharyngeal swabs for SARS-CoV-2 PCR tests. (**b**) Several fluid droplets were placed on a supporting plate. (**c**) The samples were illuminated using two halogen lamps. Sub-surface scattering is illustrated by the red arrows. (**d**) A sliding sensor recorded hyperspectral images the VNIR range. (**e**) The reflectance spectrum of each pixel within the hyperspectral matrix was then digitally pre-processed to obtain the pseudo-absorbance spectra. (**f**) Spectral features descriptors were obtained from pixel spectra for analysis. (**g**) A feed-forward neural network (FFNN) was trained^37^ to detect viral content from spectral features and output a pixel-based binary classification. (**h**) A partial least square-discriminant analysis (PLS-DA) was performed^37^ using pixel reflectance spectra.

The aim of the work presented here is to determine whether the combination of hyperspectral imaging and spectral data analysis can be used for mass screening of SARS-CoV-2. To answer this question, the optical diffuse reflectance spectra of the samples under study in the visible and near-infrared ranges were converted to pseudo-absorbance (PA) spectra and processed using spectral feature descriptors (SFDs), partial least-square discriminant analysis (PLS-DA), and machine learning (a feed-forward neural network, FFNN) to extract the information, following the methodology summarized in Fig. 1. Three different experiments were performed: Experiment 1 appraised the discernment of two types of synthetic SARS-CoV-2 models (with and without the characteristic spike protein), Experiment 2 evaluated the classification of SARS-CoV-2-positive and negative human nasopharyngeal exudate samples (the same used for conventional PCR tests), and Experiment 3 comprised an observational, descriptive study of fresh samples of SARS-CoV-2-positive and negative human saliva (see details in “Materials and methods” section and Supplementary Information).

### Spectral information from SARS-CoV-2 spike pseudotyped lentiviral particles (Experiment 1)

The first step in this study was to answer whether the PA spectra obtained from fluids containing viral particles possess sufficient information for optical detection. We compare pixel spectra obtained from droplets containing SARS-CoV-2 spike pseudotyped lentiviral particles (S-LP) with preparations of lentiviral particles pseudotyped with the G glycoprotein of the vesicular stomatitis virus (VSV-G, i.e., without the spike protein, here named as G-LP) prepared in both, phosphate buffered solution (PBS) and artificial saliva (AS), and their respective negative controls. A total of 193 droplets (corresponding to 109 G-LP, 32 S-LP and 52 controls) were analyzed (see Table 1).

**Table 1:** Experiment 1: Sample distribution of synthetic viral models (SARS-CoV-2 spike pseudotyped lentiviral particles (S-LP) and lentiviral particles pseudotyped with the G protein of the vesicular stomatitis virus (G-LP)) and their respective negative controls (S-LP Ctrl and G-LP Ctrl) in phosphate buffered solution (PBS) and in artificial saliva (AS) at droplet and pixel levels.

		PBS				AS			
		G-LP	G-LP Ctrl	S-LP	S-LP Ctrl	G-LP	G-LP Ctrl	S-LP	S-LP Ctrl
Droplet	C4	24	2	4	4	15	3	4	4
	C3	9	2	4	4	14	3	4	4
	C2	9	2	4	4	14	3	4	4
	C1	9	2	4	4	15	3	4	4
	Total	51	8	16	16	58	12	16	16
Pixel	C4	25,504	1605	4636	4725	13,381	2642	5065	5076
	C3	10,532	1600	4703	4243	12,755	2651	4535	4373
	C2	8536	1061	3701	3091	11,315	1847	4084	4534
	C1	8205	1134	3203	3519	16,594	2826	4742	4989
	Total	52,777	5400	16,243	15,578	54,045	9966	18,426	18,972

Concentrations were C1 = 800 TU µL^−1^, C2 = 1500 TU µL^−1^, C3 = 3000 TU µL^−1^ and C4 = 4000 TU µL^−1^. Data from G-LP samples were obtained from a previous study^37^.

**Figure 2:**
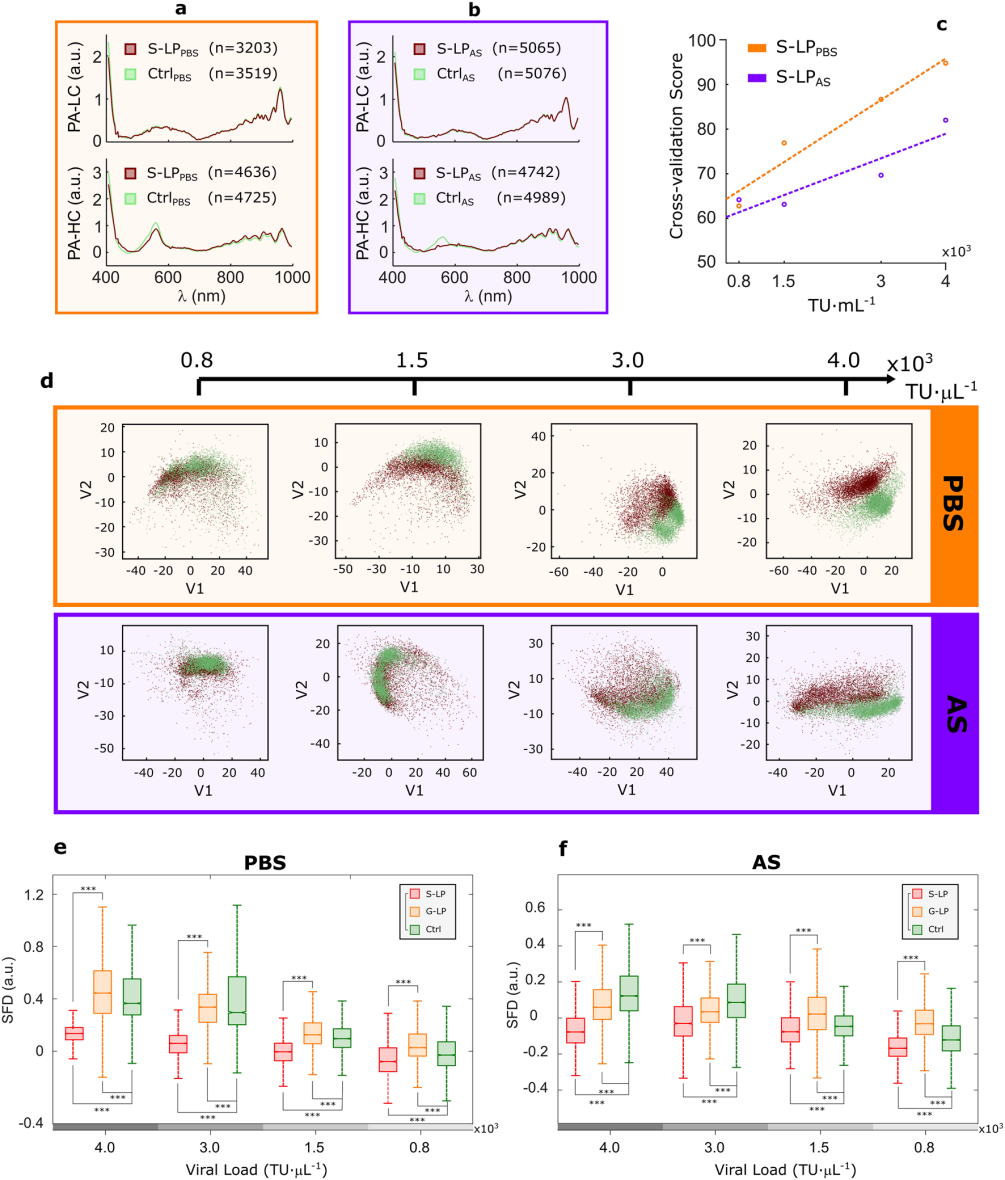
Experiment 1: Spectral discrimination of SARS-CoV-2 spike pseudotyped lentiviral particles. (**a**) Mean pseudo-absorbance (PA) pixel spectra of spike pseudotyped lentiviral particles in phosphate buffered solution (S-LP_PBS_) for high concentration (HC = 4.0 × 10^3^ TU µL^−1^) and low concentration (LC = 0.8 × 10^3^ TU µL^−1^) and their respective negative controls (Ctrl_PBS_). (**b**) Mean pseudo-absorbance (PA) pixel spectra of spike pseudotyped lentiviral particles in artificial saliva (S-LP_AS_) for high concentration (HC = 4.0 × 10^3^ TU µL^−1^) and low concentration (LC = 0.8 × 10^3^ TU µL^−1^) and their respective negative controls (Ctrl_AS_). (**c**) Overall score value of the tenfold cross-validation essay performed in partial least square-discriminant analysis (PLS-DA Model 1) in both preparations (per pixel). Dashed lines represent their linear correlations. (**d**) Scatter plot of the two latent variables (V1 and V2) used in the PLS-DA Model 1 for different viral concentrations. Brown and green dots represent positive and control samples respectively. (**e,f**) Spectral feature descriptor (SFD), computed in the band 483–610 nm for S-LP and G-LP samples in phosphate buffered solution (PBS), artificial saliva (AS), and the corresponding culture media as control (Ctrl). ***p-value = 0 using the Wilcoxon rank sum test. Outliers were removed for clarity.

These viral particles are comparable to the SARS-CoV-2 in overall shape and diameter, have a double lipid envelope and were engineered to also resemble the molecular structure of the surface of the SARS-CoV-2 virions, characterized by the protruding spike proteins. They allowed for a preliminary assessment of our approach while reducing the biosafety requirements needed to handle SARS-CoV-2 samples^52^. Differences were observed in the overall PA spectra obtained from samples carrying the virus and their negative controls, as shown in Fig. 2a,b. These differences appeared more evident at higher viral concentrations. We performed a partial least square-discriminant analysis to reduce the intrinsic complexity of large subsets of pixel spectra using two latent variables and explore the differences among positive and negative pixels, as illustrated in Fig. 2d. The performance score obtained from a stratified tenfold cross-validation (Fig. 2c) showed that there was sufficient information to differentiate the individual pixel spectra obtained from preparations with viral particles from those used as a negative control. In fact, the performance score showed a very strong linear correlation (r2 = 0.95) with the viral concentration in PBS preparations, and a strong correlation (r2 = 0.84) for preparations in AS (Fig. 2c). Note that the viral concentrations used here were in the range of those found in expelled respiratory fluids from SARS-CoV-2 positive cases^53^. These findings suggested that this method may be applied to detect the intended pathogen in human specimens.

Body fluids such as saliva contain a wide variety of biomolecules and might even carry other viral species. Consequently, we sought to find whether two similar viral particles of the same type, shape and size could be distinguished based only on the presence of the characteristic spikes. We calculated a spectral feature descriptor (namely, SFD) as a measurement of the relative difference between the diffuse reflectance of the sample under analysis and that of its supporting plate over a certain wavelength range, in this case between 483 and 610 nm. We then compared this spectral descriptor from S-LP samples with G-LP preparations obtained in a previous study^37^ (see Table 1). Normality of the distribution was discarded using the Kolmogorov–Smirnoff test. The Wilcoxon rank sum test performed on the values of the said descriptor demonstrated statistically significant differences (p-value = 0) between both viral preparations and the controls for each concentration, as shown in Fig. 2e,f. It must be noted that the differences in the spectral descriptor are more evident at higher viral concentrations, with no overlapping between the inter-quartile ranges from both viral preparations (particularly in PBS). These differences point at the molecular composition of the virions, related to the proteins integrated in their membrane.

Findings in this section suggest that our proposed method could be used to discern the presence of the SARS-CoV-2 spike protein in viral solutions in the synthetic biofluids under study (saline solution and artificial saliva). Further analysis could help to determine whether the spectral feature descriptor represents a structural or molecular difference between viruses or a different interaction of the virus with the medium in the fluid.

### Detection of SARS-CoV-2 in nasopharyngeal exudates (Experiment 2)

We also sought to answer whether the proposed optical technique could be used for the analysis of inactivated nasopharyngeal exudate specimens employed for PCR testing. Typically, the transport media in the commercial swabs contain a lysis buffer such as guanidinium thiocyanate which inactivates the viral particles while preserving the genetic material^54^. Hence, the molecular structure of the viral particles is not maintained and therefore, the optical information embedded in these preparations may differ. As previously determined, the optical technique described here can obtain virus-specific information related to the proteins integrated in the viral capsid. To test whether optical detection under safer conditions is feasible, we analyzed inactivated nasopharyngeal exudates from 72 subjects—including 31 positive (24 men, 6 women, and 1 pediatric female case) and 41 negative (16 men, 23 women, and 1 pediatric male and 1 pediatric female cases)—using the same two pixel-based classification methods, i.e., PLS-DA and FFNN.

Following the same classification strategy described in a previous publication^37^, PLS-DA Model 2 and the FFNN model were built from individual pixel data. Their outputs provided per-pixel classifications, subsequently integrated at droplet and patient levels to enhance the mutual information contained in the viral debris. An additional PLS-DA Model 3 was built using patient-averaged spectra, and its output provided a direct per-patient classification (on the same patient sets). Note that training sets employed for PLS-DA models combine the training and validation sets used for the FFNN, while test sets are the same for both classification procedures. The total number of patients was randomly split in a fourfold cross validation of the FFNN. Table 2 shows the sample distribution for Trial 1 (see Supplementary Information for Trials 2, 3 and 4).

**Table 2:** Experiment 2: Sample distribution of nasopharyngeal exudates at different levels (total numbers of patients, droplets and pixels) among the experimental Training (Tra), Validation (Val), and Test groups (Trial 1).

		Pixel				Droplet				Patient				
		*Pos*			*Neg*	*Pos*			*Neg*	*Pos*				*Neg*
		H	M	L		H	M	L		H	M	L	*Total*	
PLS-DA	*Tra*	144,426	35,715	30,545	808,184	78	24	18	150	13	4	3	20	25
	*Test*	50,264	32,902	26,579	146,440	30	18	18	96	5	3	3	11	16
FFNN	*Tra*	111,676	27,924	21,498	744,855	60	18	12	108	10	3	2	15	18
	*Val*	32,750	7791	9047	63,329	18	6	6	42	3	1	1	5	7
	*Test*	50,264	32,902	26,579	146,440	30	18	18	96	5	3	3	11	16

Positive (Pos) and negative (Neg) cases were determined by qRT-PCR. Positive cases include three viral load levels (H = 10^6^ copies mL^−1^, M = 10^4^ copies mL^−1^ and L = 10^2^ copies mL^−1^). Note that classification algorithms provide results as (per-pixel, per-droplet and per-patient) ‘positive’ (with any level of viral load) or ‘negative’ assignations.

*PLS-DA* partial least square-discriminant analysis, *FFNN* feed-forward neural network.

The spectral feature descriptor (SFD) was also computed in a band with visible differences among averaged PA spectra (between 870 and 910 nm) to analyze the viral load of samples. Positive cases were confirmed by quantitative reverse transcription PCR (qRT-PCR) in three levels of viral load, high (H: 10^6^ copies mL^−1^), medium (M: 10^4^ copies mL^−1^) and low (L: 10^2^ copies mL^−1^). Table 2 shows the sample distribution among the different experimental groups.

**Figure 3:**
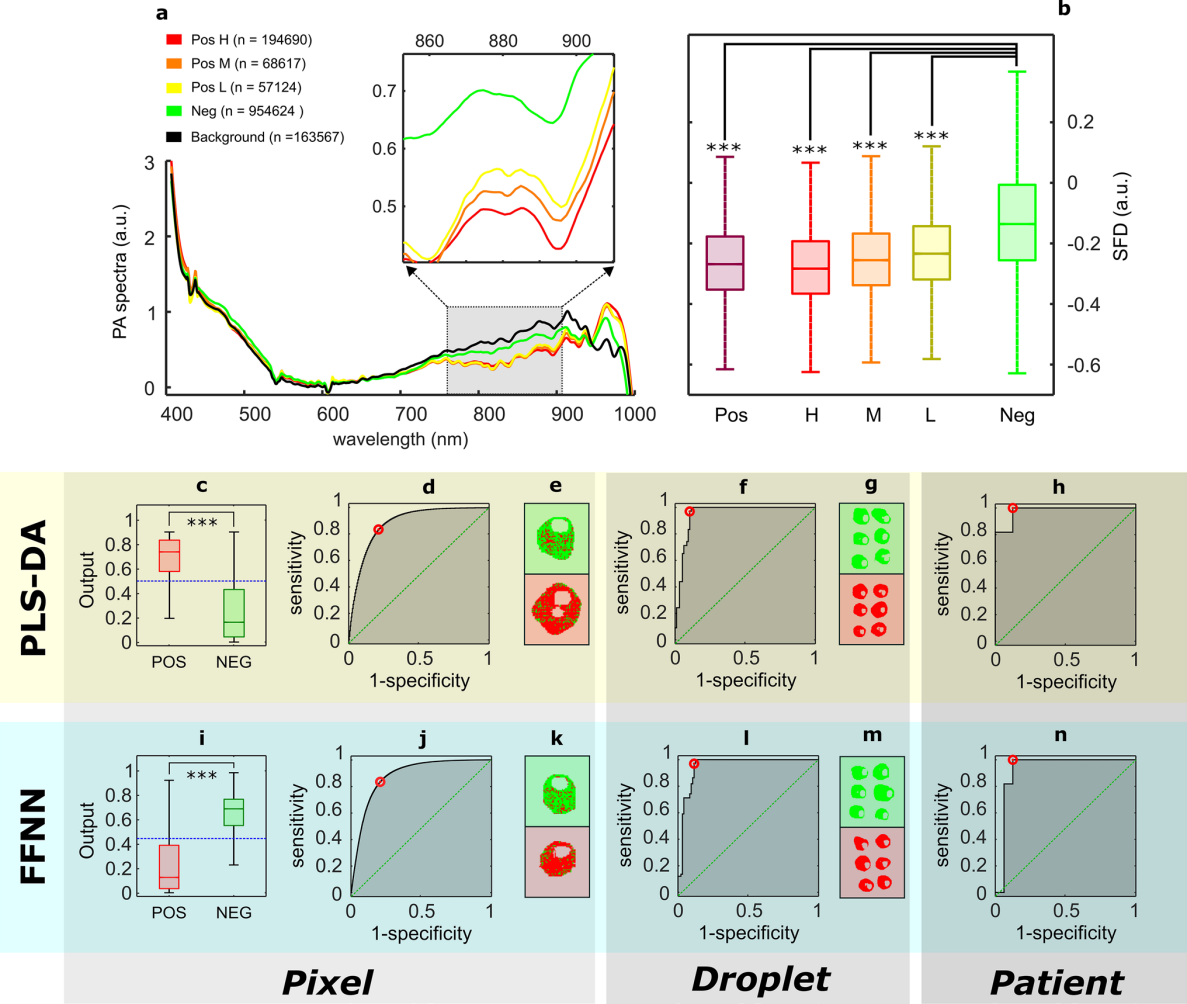
Experiment 2: Analysis of SARS-CoV-2 nasopharyngeal exudates. (**a**) Mean pseudo-absorbance (PA) pixel spectra from positive (viral loads H = 10^6^ copies mL^−1^ (high), M = 10^4^ copies mL^−1^ (medium) and L = 10^2^ copies mL^−1^ (low)) and negative cases of SARS-CoV-2. In addition, the panel shows the mean pixel spectra of the supporting plate (background). (**b**) Value of the spectral feature descriptor (SFD) in the band between 870 and 910 nm for all cases. Positive (Pos) box includes all H, M and L samples. (**c–h**) Classification results obtained using partial least square-discriminant analysis (PLS-DA Model 2, Trial 1). (**i–n**) Classification results obtained using a feed-forward neural network (FFNN, Trial 1). (**c,i**) Median value of the output used for pixel classification. Positive and negative samples were determined by qRT-PCR. ***p-value = 0 using Wilcoxon rank-sum test. The blue dashed line illustrates the classification threshold. (**d,j**) Receiver operating characteristic (ROC) curves from the pixel classification. (**e,k**) Example of the resulting pixel classification in a droplet. Red and green pixels were classified as positive and negative respectively. Note red and green backgrounds indicate a positive sample and its negative control. (**f,l**) ROC curves obtained from droplet classifications. (**g,m**) Example of the classification of several droplets from a positive (red background) and a negative (green background) patient. (**h,n**) ROC curves obtained from patient diagnosis. Outliers were removed for clarity.

Figure 3a shows their mean pseudo-absorbance pixel spectra and Fig. 3b the value of the spectral feature descriptor (SFD) in the band between 870 and 910 nm. Figure 3c–h shows the classification results obtained by the PLS-DA Model 2 (Trial 1), and Fig. 3i–n the results given by the FFNN (Trial 1, see Supplementary Information for Trials 2, 3 and 4). Classification results are shown as receiver operating characteristic (ROC) curves with the corresponding values of the area under the curve (AUROC).

As we anticipated, the differences in the PA spectra obtained in Experiment 2 (nasopharyngeal exudate samples in the inactivation medium) were less informative than those obtained from the previous preparations of Experiment 1 (synthetic viral models in saline solution and in artificial saliva).

The per-pixel PLS-DA Model 2 was built using the samples from 45 patients (20 qRT-PCR-positive, 25 qRT-PCR-negative). The test group included 27 subjects (11 positive and 16 negative) as described in Table 2. A total of 12 latent variables were used, and the variance captured was 95.15%. The outputs of the PLS-DA were used to classify the individual pixel spectra. After discarding normality, the two-tailed Wilcoxon rank-sum test performed on the classification output showed statistically significant differences between positive and negative samples (p-value = 0), as shown in Fig. 3c. The ROC curve was constructed to determine the pixel classification cut-off value that optimized both, sensitivity and specificity (Fig. 3d,e). The obtained AUROC showed a good accuracy (AUROCpixel = 0.88). However, a noticeable improvement was observed by integration at droplet level (AUROCdroplet = 0.95), as shown in Fig. 3f,g. Finally, at patient level, we found a very good agreement between the qRT-PCR assays and our proposed technique (sensitivity = 100%, specificity = 87.5%), as illustrated in Fig. 3h.

Next, the same pixel PA spectra were classified using a FFNN. Following the procedure described in a previous work^37^, the input of the network was a set of 28 spectral shape features computed in four spectral fringes, and the output provided a pixel-level binary classification. In this case, the neural network was trained using samples from 33 patients (15 qRT-PCR-positive, 18 qRT-PCR-negative), validated using samples from 12 patients (5 qRT-PCR-positive, 7 qRT-PCR-negative), and tested in the same subset of 27 patients used in the per-pixel PLS-DA Model 2, as summarized in Table 2. Note that both test and validation splits were randomly assigned from the training group used in the PLS-DA Models 2 and 3. Similarly, after rejecting normality, the Wilcoxon rank-sum showed statistically significant differences in the output of positive and negative pixels (p-value = 0), as illustrated in Fig. 3i. The performance of the classifier, as in the case of the PLS-DA, improved importantly from pixel level (AUROCpixel = 0.88) to patient level (AUROCpatient = 0.93), as shown in Fig. 3j–n. The performance of both classification methods was comparable in terms of sensitivity and specificity at all levels under consideration, as shown in Table 3. Additionally, we performed a fourfold cross-validation essay with random splits (over patients, see Supplementary Information), obtaining an overall sensitivity and specificity of 94.7 ± 4.6% (mean ± std) and 92.7 ± 2.8% (mean ± std) (see Supplementary Information). The overall AUROC was 0.95 ± 0.01 (mean ± std).

**Table 3:** Experiment 2: Values of sensitivity (SE), specificity (SP) and area under the receiving operating characteristic (AUROC) curve obtained at per-patient classification from both types of analysis, partial least square-discriminant analysis (PLS-DA Models 2 and 3) and the feed-forward neural network (FFNN), of inactivated nasopharyngeal exudates using the same patient sets (Trial 1).

	PLS-DA (Model 2) (per-patient, from per-pixel classification)			FFNN (per-patient, from per-pixel classification)			PLS-DA (Model 3) (per-patient, from patient-averaged spectra)		
	SE (%)	SP (%)	AUROC	SE (%)	SP (%)	AUROC	SE (%)	SP (%)	AUROC
Pixel	83.3	78.8	0.88	83.5	79.2	0.88	–	–	–
Droplet	97.0	89.6	0.95	97.0	88.5	0.95	–	–	–
Patient	100.0	87.5	0.98	100.0	87.5	0.93	90.9	87.5	0.97

The additional PLS-DA Model 3 was built upon the averaged pixel spectra per patient for comparison (see Supplementary Information). Its overall sensitivity and specificity were 90.9% and 87.5% respectively, in very good agreement with the results provided by the per-pixel models given by PLS-DA Model 2 and by the FFNN (see Table 3, Supplementary Information).

Findings in this section show that proposed optical analysis could classify positive and negative cases of SARS-CoV-2 using the same inactivated nasopharyngeal exudates than conventional PCR tests.

### Spectral information from fresh saliva from SARS-CoV-2 patients (Experiment 3)

Human saliva has been proved to be a body fluid suitable for detecting ongoing infections of SARS-CoV-2^55–57^. Therefore, we analyzed the optical PA spectra obtained from saliva specimens to determine if we could extract useful information using the proposed technique.

As indicated, data collection for this study was carried out during the first wave of the COVID-19 pandemic. Under such extraordinary conditions, it was very difficult to obtain and manage SARS-CoV-2 positive samples. Based on the results described in previous sections, we limited our analysis of fresh saliva samples to an initial, descriptive study with a reduced number of cases to evaluate the feasibility and potential interest of our technique for this application.

**Figure 4:**
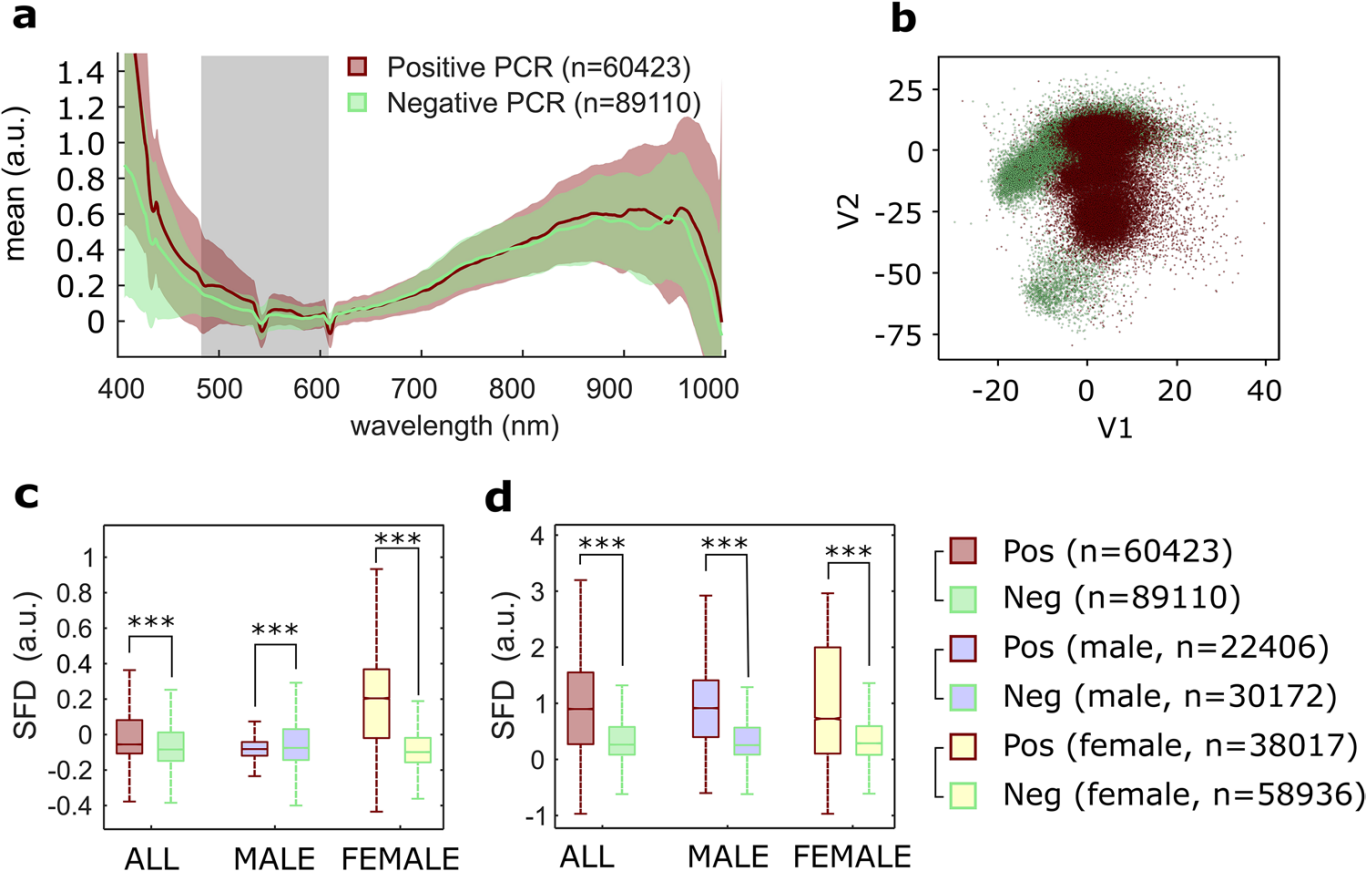
Experiment 3: Spectral information from SARS-CoV-2 in fresh saliva specimens. (**a**) Mean (± std) of the pseudo-absorbance pixel spectra from fresh saliva samples. Positive (pos) and negative (neg) cases were determined by PCR test. (**b**) Scatterplot of the two latent variables obtained in partial least square-discriminant analysis (PLS-DA) Model 1 from the pixel spectra. Positive and negative PCR tests are brown and green dots respectively. (**c**) Value of the spectral feature descriptor (SFD) obtained between 483 and 610 nm (grey band in (**a**)) for positive and negative cases. (**d**) Value of the spectral descriptor SFD obtained between 407 and 470 nm for positive and negative cases. (**c,d**) Both panels include differentiation between male and female pixel sets. ***p-value = 0 using the Wilcoxon rank sum test. Outliers were removed for clarity.

Under biological containment conditions equivalent to biosafety level 3, we studied the pixel spectra obtained from a total of 192 droplets from 6 positive (3 men and 3 women) and 26 negative (8 men and 18 women) cases determined by PCR test. These sets are clearly imbalanced with respect to the presence of SARS-Cov-2 and gender. Nevertheless, differences were observed in the PA spectra of positive and negative cases, as illustrated in Fig. 4a.

To explore those differences, a PLS-DA Model 1 (using all samples) was constructed upon two latent variables and the clusters are shown in Fig. 4b. The spectral feature descriptor was also computed in the range between 483 and 610 nm (Fig. 4c) and between 407 and 470 nm (Fig. 4d). Normality was discarded using the Kolmogorov-Smirnoff test. The Wilcoxon rank sum test revealed statistically significant differences in the values of the SFD (p-value = 0).

Despite the relatively low number of positive cases presented in this section, these findings suggest that the method presented here might be used to differentiate the presence of the SARS-CoV-2 in human fresh saliva samples. We also found statistically significant differences in the values of the spectral feature descriptor for positive and negative cases, both for male and female samples (Fig. 4c,d, p-value = 0).

## Discussion

The development of fast and reliable screening techniques represents a critical enabler in a pandemic scenario, as quick, easy-to-implement and cost-effective test methods are essential for the detection of positive cases to curb propagation and to provide viral prevalence data^58^. In the specific context of the COVID-19, given the implications of false negatives for the spread of the disease^59^, highly sensitive tools are urgently needed for mass screenings to identify virus carriers^58,60^, even at the cost of reduced specificity relative to other respiratory viral species. In resource-constrained settings such screening approaches may also be useful to protect high-risk patients in those scenarios^12^.

Here we have demonstrated the strong potential of hyperspectral image analysis in the visible and near-infrared range to contribute to fighting the transmission of the SARS-CoV-2, as it allows for high-throughput testing by scanning many samples simultaneously using standard, relatively simple, optical equipment. This technique does not require the addition of any reagent to the samples or the amplification of nucleic acids, and may therefore be easily deployed in transport hubs, mass events, and during vivid outbreaks.

In this study the detection and classification results of exudate samples were obtained applying two different and independent analytical procedures: partial least square-discriminant analysis and feed-forward neural networks. PLS-DA is a statistical multivariate data processing technique commonly employed for descriptive analysis and predictive classification of highly dimensional data, including genomic data sets^61^, metabolomics^62^ and hyperspectral images in a variety of applications, from airborne remote sensing for precision agriculture^63^ and land monitoring^64^ to ground-based implementations for food inspection^65,66^, characterization of materials^67^ and many others. As compared to other approaches (e.g., principal component analysis, PCA), PLS-DA methodology provides dimension reduction for per-pixel classification in image processing^68^. FFNNs belong to the powerful set of machine learning algorithms, within the wide field of artificial intelligence. They are also usually applied for hyperspectral image analysis in computer vision systems^69^.

### Conceptual assessment

The results of Experiment 1 show that proposed technique effectively achieves the differentiation of the synthetic viral models—with and without the characteristic spike protein of SARS-CoV-2—at the four tested concentrations (Fig. 2a–d). Their relative differences (between samples with S-LP and G-LP) increase in accordance with higher viral loads in both a ‘simple’ fluid (PBS, a type of saline solution) and in the much more complex (realistic) artificial saliva (Fig. 2e,f). These results expand those reported in a previous study^37^ on the detection and quantification of G-LP models as liquid droplets and dry residue in the same fluids using the proposed technology. It is therefore considered that the ‘differential element’ in all tests performed on synthetic viral models, i.e., the presence of the SARS-CoV-2 Spike protein in the viral particles (and not the protein alone), would trigger the observed detection and constitute the conceptual basis for the subsequent analysis of human samples with the actual SARS-CoV-2.

A similar appraisal refers to results obtained from human samples of nasopharyngeal exudate in Experiment 2. The specimens were obtained in a small geographical area during a relatively short period of time from subjects who experienced COVID-19 compatible symptoms. As described in the methodology, positive cases were confirmed using a qRT-PCR test, and therefore, symptoms in the negative cases had to be caused by any of the other seasonal respiratory pathogens in circulation^70,71^. It is known that the vast majority of respiratory infections are caused by a virus^72^, therefore, the sensibility and specificity obtained here reflects the ability of this technique to discriminate between SARS-CoV-2 and other respiratory viral species present in the geographical location of sampling during the study period (most likely, influenza and common cold), and further studies of the specificity of detection versus those species remain to be developed. Despite the genetic diversity of SARS-CoV-2^73,74^, the achieved sensitivity for analysis of exudate samples is comparable to those of other rapid methods. It is important to stress the high level of agreement (Table 3) among the classification results for exudates obtained from the three different, independent numerical procedures applied to the same sets of patients: (i) a per-pixel PLS-DA model (PLS-DA Model 1)—later integrated to per-patient level—whose inputs were the individual spectra of all sample pixels, (ii) a FFNN per-pixel model built from a set of numerical shape descriptors calculated in certain spectral fringes of interest, also integrated to per-patient level, and (iii) a PLS-DA model directly constructed at per-patient level (PLS-DA Model 3), whose inputs were the patient-averaged spectra. Remarkably, we achieved a sensitivity of 100%, with a very high overall classification accuracy of the same exudate samples used for standard PCR tests, using both PLS-DA and FFNN methods, in a study group which included patients with low viral loads determined by qRT-PCR.

In Experiment 3, an observational, descriptive study of samples of fresh human saliva was performed. Differences were obtained in the PA spectra of positive and negative cases (Fig. 4a). Although the number of cases did not allow for a per-patient classification, a tenfold cross validation showed a high overall accuracy (87%) for individual classification of pixels belonging to positive and negative patients. We also observed differences in the values of this descriptor between male and female cases. These early findings agree with the sex differences observed in the severity and evolution of the disease^75^. Additional essays should consider those factors, as well as whether smoking, food and drink intake, or pathologies that may alter the composition and properties of saliva or nasopharyngeal exudates have undesirable effects on the detection accuracy.

### Sample sizes and data processing

This study presents the analysis of samples at different levels (per-pixel, per-droplet and per-patient), according to the experiments. Training and validation sets for the FFNN model were combined as the training set for the PLS-DA models, while tests sets were identical. Note that random splits of independent samples were constructed on a per-droplet (in Experiment 1) and on a per-patient basis (in Experiments 2 and 3), to avoid mixing of pixels from a given droplet (in Experiment 1) or from a patient (in Experiments 2 and 3) in different sets. All pixels belonging to the same patient are related and cannot be considered as fully independent samples, however potential unwanted effects due to specific or physiologic features of individual subjects or to eventual impurities or incidences evading quality controls in the fabrication processes of synthetic models would be cancelled by random splits of samples in the k-fold cross-validation procedures performed.

In classification analyses, the category of ‘positive’ samples included cases with the three levels of viral load considered. Therefore, in the per-pixel approach (using PLS-DA Model 2 and the FFNN model) the number of pixels in each training, validation and test set was in the order of several (tens of) thousands (Table 2). In the classification of exudate samples (Experiment 2) an additional model (PLS-DA Model 3) was independently built on a per-patient basis, as each subject was characterized by a single spectrum resulting from averaging all individual spectra of pixels (from all 6 droplets).

The number of latent variables of PLS-DA Models 2 and 3 was determined to retain over 95% of data variance providing optimal discrimination of cases in each (positive and negative) class. The number of free parameters (neurons) of the FFNN can estimated within the range given by the product of number of neurons per layer and the number of layers, i.e., 20 × 112 = 2240 parameters (sparsely connected), and 112^20^ parameters (fully connected). This value is much higher than the number of independent sample subjects but, for sparse connection, much smaller than the number of sample pixels in each set. As indicated, the per-patient PLS-DA Model 3 was built and tested on the same training and test sets as the per-pixel PLS-DA Model 2 and the FFNN, and the results of the three procedures agreed. SFD was calculated for all pixels and represented in boxplots to analyze the viral loads in the three concentrations tested.

Limitations of our study also include the use of relatively reduced groups of cases. In the analysis of exudate samples, a larger set is required for proper comparison of accuracy with existing technologies previously indicated (e.g., Raman spectroscopy, mass spectrometry), while in the assessment of fresh saliva, the patient set employed is not sufficient to perform the same classification as with exudates. Analyzed samples were also partly unbalanced regarding sex and viral load, and other factors which may relate to the COVID-19 progression in patients (e.g., age, body mass index, disease variants) were not considered.

### Technological assessment

As indicated, currently available optical strategies for the detection of COVID-19 mainly rely on microscopy imaging set-ups and on lab-on-chip approaches. They analyze a very small linear field-of-view (FOV), with a very reduced working distance (WD) between the optical device and the sample, commonly using specific (e.g., laser) light sources. On the contrary, our approach relies on an imaging configuration that registers a relatively wide FOV, at a WD in the order of tens of centimeters, under broadband illumination from conventional halogen sources. This scheme allows for simultaneous scanning of multiple droplets simply deposited on a supporting surface, following a procedure which can be easily scaled up to increase throughput.

Performance at the point-of care can be easily up-scaled. Our current setup registers a FOV of 7 × 10 cm^2^ at a WD of 30 cm, scanning samples from one patient (i.e., six 5-µL droplets) in 40 s; however, with the same configuration and resolution, it could scan up to 54 patients in 300 s. In addition, the proposed setup is easy to install, and facilitates a safe manipulation of the samples by minimally trained operators. Under the extraordinary circumstances of the first pandemic outbreak, we used commercial components and a high-level programming language to develop a prototype for standard personal computers. The total sample-to-result time interval for classification of a given patient (using 6 droplets) was eight minutes for PLS-DA methods, and five minutes for the FFNN algorithm. Processing times can be substantially reduced to a few seconds by adjusting the size and number of droplets per case to be analyzed, and by optimizing of the classification algorithms, for example, by using parallel computing techniques to process each pixel spectrum independently. It is important to consider that while the use of inactivated samples reduces biological hazards, it also increases the acquisition time, as it requires a trained health care professional to obtain the specimens.

The proposed methodology comprises two different processing stages: the generation of numerical models for classification (using droplets of known diagnosis to train the system), and their application for classification. As described, numerical models can be developed using two approaches: advanced statistical tools (PLS-DA methods) and artificial intelligence (machine learning FFNNs). While neural networks require larger, balanced, training and validation sets, they offer, in turn, the potential ability of substantial improvement in performance following artificial intelligence (e.g., machine-learning) procedures, effectively tackling the inherent variability of human biology and individual circumstances.

From an engineering point of view, the generation of numerical models and the training of artificial neural networks are time-consuming processes. However, these models can be quickly improved and updated and, when accomplished, can be applied for the simultaneous classification of all samples within the registered field of view of the imaging system. Thus, assuming approximately a thousand pixels per droplet (and six droplets per patient) an individual test result could be obtained within few seconds after image acquisition.

Given that a single hyperspectral image can include samples from several patients, our method would enable concomitant, ultra-fast screening with sample-to-result time under one minute per patient, well below other rapid tests, including optical and spectroscopic technologies. In addition, our system may be extended to include different viral species by re-building and updating the numerical models with enlarged data sets. Furthermore, the optical equipment for image acquisition can be further integrated into a computer-controlled device, suitable for use by personnel with minimal training. Recent advances in optical adapters for spectroscopy^76^ using smartphones might even allow for its implementation in personal, mobile devices.

Limits of detection determine the suitability of a given technology for the intended application. In this work, synthetic SARS-CoV-2 models (Experiment 1) were prepared in the same concentrations as in a previous study^37^ to facilitate their direct comparison, from 800 TU µL^−1^ to 1500 TU µL^−1^, 3000 TU µL^−1^ and 4000 TU µL^−1^. If translated into the equivalent ‘physical units’ (copies mL^−1^), assuming a 0.1% of infectious particles in the samples^77^, that lowest value corresponds to 8 × 10^8^ copies mL^−1^. SARS-CoV-2 positive human samples of exudate (Experiment 2) were classified by qRT-PCR in three levels of viral load, high (10^6^ copies mL^−1^), medium (10^4^ copies mL^−1^) and low (10^2^ copies mL^−1^), and human samples of fresh saliva (Experiment 3) were classified by qualitative PCR as SARS-CoV-2 positive/negative, without quantification.

Concentration values of physiological interest lie below the sub-picomolar range^32^. Interestingly, the average viral load of COVID-19 patients is^53^ about 7 × 10^6^ copies mL^−1^, approximately equivalent to a 0.01 pM concentration, while the lowest viral load of exudate samples analyzed here was 10^2^ copies mL^−1^. In addition, the estimated viral load of individuals^45^ considered as ‘super-carriers’ of SARS-CoV-2 corresponds to the lowest concentration employed for the analysis of synthetic models.

### High potential

We have introduced the use of hyperspectral image processing for the detection of SARS-CoV-2 in clinical samples. The results here support its ability to analyze inactivated nasopharyngeal swabs to detect SARS-CoV-2 and discern positive carriers from other patients with respiratory symptoms, and its ability to differentiate SARS-CoV-2 from other viruses is still to be fully determined.

Our approach could also be applied to saliva samples. Although they present an inherent handling risk, collection can be performed by the subjects themselves without requiring the use of a swab^57^. Note that the number of salivary specimens analyzed here was not large enough to perform a classification assessment. Nevertheless, there are three outcomes here, obtained using different analytical approaches that altogether suggest saliva could be used as well. First, we found substantial optical information arising from the spike protein that conforms the viral capsid of the engineered lentiviral particles. Secondly, an accurate classification was achieved using inactivated samples despite the structural alteration of the viral capsid caused by the inactivation medium (this aspect and the effects of different commercial buffers, remain to be studied in further analysis). And thirdly, we could establish significant differences between positive and negative cases from saliva samples. It must be highlighted that values of the spectral feature descriptor calculated from individual pixel spectra—independently of PLS-DA and FFNN models—increase (lineally) according to the concentration of viral models in biofluids and of SARS-CoV-2 inactivated exudate samples, also showing differences—in coincidence with other reported analysis—between fresh saliva of male and female SARS-CoV-2 positive and negative subjects (although in a limited number of cases). This relationship between values of a spectral descriptor and the viral load of samples supports the hypothesis of the underlying information embedded in sample pixel spectra.

The presented approach relies on the analysis of samples using numerical models, which can easily be improved and updated with larger datasets. This technology is reagent-free and easy to implement and scale-up—even in resource-limited scenarios—for high-speed, high-throughput testing, as it allows for the simultaneous analysis of many samples (namely, all droplets within the field of view registered by the camera) using relatively common imaging equipment, suitable for safe use by minimally skilled operators. The described methodology could substantially improve the workflow in a primary screening stage, and be implemented at the point-of-care to select those subjects who should undergo further evaluation^78^. This use would allow for a relevant reduction in the detection time, logistics and costs associated with molecular analysis.

Overall, our findings support the use of hyperspectral image analysis for rapid, large-scale, high workflow SARS-CoV-2 primary screening at the point-of-care under the currently demanding circumstances of the COVID-19 pandemic. However, further studies with larger patient cohorts are required for refining the sensitivity and specificity of the technique. The proposed use of numerical models may also open the path for other applications to be developed.

## Materials and methods

### Experimental protocols

**Figure 5:**
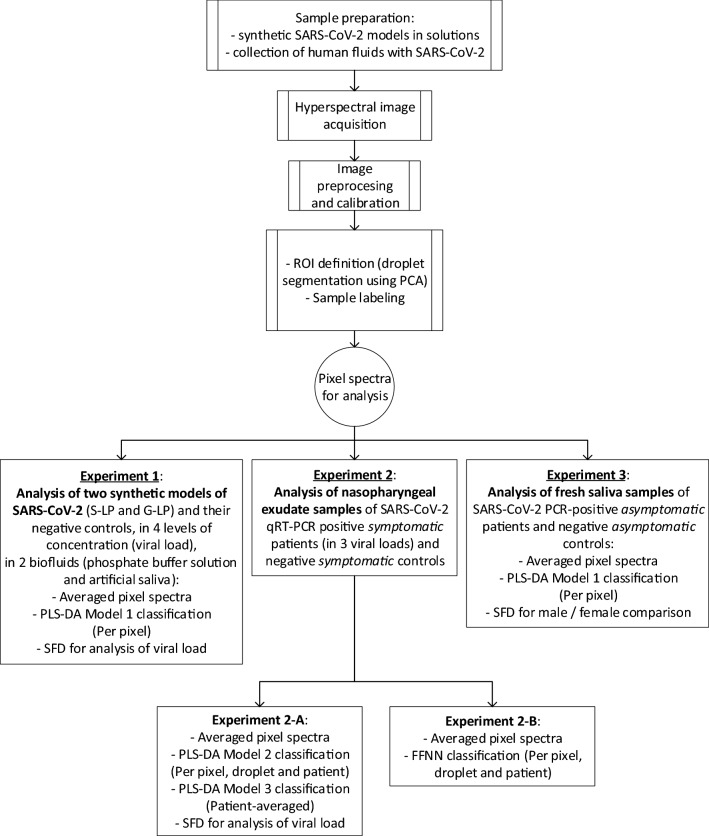
Summary of the three performed experiments. Experiment 1: Analysis of two synthetic models of SARS-CoV-2 (S-LP and G-LP) and their negative controls, in four levels of concentration (viral load), in two biofluids (phosphate buffered solution and artificial saliva). Experiment 2: Analysis of nasopharyngeal exudate samples of SARS-CoV-2-positive symptomatic patients (in three viral loads) and negative symptomatic controls. Experiment 3: Analysis of fresh saliva samples of SARS-CoV-2-positive asymptomatic patients and negative asymptomatic controls. *ROI* region of interest, *PCA* principal component analysis, *S-LP* lentiviral particles pseudotyped with the SARS-CoV-2 Spike protein, *G-LP* lentiviral particles pseudotyped with the vesicular stomatitis virus G protein, *PLS-DA* partial least square-discriminant analysis, *FFNN* feed-forward neural network, *SFD* spectral feature descriptor, *qRT-PCR* quantitative reverse transcription polymerase chain reaction (PCR).

The approach described herein was tested from different, complementary points of view in the three independent experiments detailed in Fig. 5 and in the Supplementary Information.

### SARS-CoV-2 spike pseudotyped lentiviral particles

Lentiviral particles pseudotyped with the S-protein of the SARS-CoV-2 (for Experiment 1) were prepared as described in a previous study^37,79^. Briefly, HEK293T cells were cultured in Gibco Dulbecco’s Modified Eagle Medium (DMEM). The culture medium was supplemented with 10% fetal bovine serum (Biowest, Nuaillé, France), and a mixture of penicillin (100 UI mL^−1^) and streptomycin (100 μg mL^−1^) (MilliporeSigma, Missouri, USA) was added to prevent bacterial contamination. The HEK293T cells (packaging cell line) were transfected with a lentiviral plasmid encoding the SARS-CoV-2 spike protein for the envelope and the plasmids encoding the genes required in a 2nd-generation lentiviral system including capsid, integrase and retrotranscriptase (Tat, Gag-Pol and Rev) as well as with a third vector coding for ZsGreen fluorescent protein flanked by long terminal repeats and packaging signals that will cause the ribonucleic acid to be packed inside viral particles. The cells were then incubated for 48 h, afterwards the supernatant was collected, and Lenti-X concentrator (Takara Bio Inc., Shiga, Japan) was added to precipitate viral particles. The mixture was then centrifuged, the supernatant was removed, and the resulting precipitate was stored at − 80 °C. As a negative control, the same number of HEK 293T cells were cultured with no plasmids, and the medium was collected and precipitated as described above. The experimental samples were prepared from a resuspended viral aliquot (or the equivalent negative control) in 1700-0305 artificial saliva (Pickering Laboratories, California, USA) or phosphate buffered saline (MilliporeSigma, Missouri, USA), an isotonic solution containing 137 mM NaCl, 2.7 mM KCl, 10 mM Na2HPO4, and 1.8 mM KH2PO4, one hour before hyperspectral imaging. Sample concentrations were 800 TU µL^−1^, 1500 TU µL^−1^, 3000 TU µL^−1^ and 4000 TU µL^−1^.

### Vesicular stomatitis virus G (VSV-G) protein pseudotyped lentiviral particles

We used data from a previous paper^37^. Lentiviral particles pseudotyped with the VSV-G protein (for Experiment 1) were prepared following the same procedure^65^ and in the same concentrations as in this work.

### SARS-CoV-2 specimen collection

The study was approved by the regional Research and Ethics Committee of University Hospitals ‘Virgen Macarena and Virgen del Rocío’, Seville, Spain (reference 0945-N-20). Prior to enrolling, all participants or their legal guardians read and signed an informed consent form. All procedures and methods were implemented according to the guidelines and regulations of the Declaration of Helsinki.

Nasopharyngeal exudates (for Experiment 2) were collected from 72 subjects (40 men and 29 women, age range of 20–90 years, and 3 pediatric cases (1 male, 2 female), age range 7–14 years) referred by a general practitioner as suspected cases of COVID-19 between April and June 2020 in Seville, Spain. During this interval, there was a strict lockdown, and geographic mobility was severely restricted. Specimens were placed within inactivating viral transport medium (Biocomma, Shenzhen, China) before processing. All the nasopharyngeal samples were analyzed following a standard qRT-PCR testing procedure. The viral RNA was extracted using a Patho Gene-spin® DNA/RNA extraction kit (Intron Biotechnology, Gyeonggi, South Korea). Then, a quantitative real-time reverse-transcriptase PCR (qRT-PCR) assay for the RNA-dependent RNA polymerase gene was performed using a qRT-PCR kit (NZYTech, Lisbon, Portugal). The specimens were considered positive if the cycle threshold (Ct) value was below 37, and negative otherwise. Positive cases were classified in three levels of viral load, high (H: 10^6^ copies mL^−1^), medium (M: 10^4^ copies mL^−1^) and low (L: 10^2^ copies mL^−1^). All negative cases were symptomatic, with respiratory symptoms compatible with the infection by SARS-CoV-2.

Fresh saliva samples (for Experiment 3) were collected from 32 adult donors (11 men and 21 women, age range of 19–101 years) were processed within 3 h from collection time. A 6-h fasting period (including oral medication) was required prior to donation. A PCR test was performed to identify positive and negative cases. All positive cases were symptomatic, and all negative cases were asymptomatic, as evaluated by a general practitioner. Smokers and subjects with diseases that might alter the composition of saliva (e.g. diabetes mellitus) were excluded from the studio. Samples with mucus were discarded from optical analysis.

It must be noted that the sensitivity of PCR tests is mainly determined by the number of amplification cycles (namely, the cycle threshold (Ct) value) required to detect the viral nucleic acids in the sample. If their concentration (i.e., the viral load) is low, more cycles are required for detection. However, there is no common, precise equivalence of Ct values to viral concentration since the process of analysis is highly dependent on the specific reagents and the technology employed and standards of know concentration are not used. Each testing unit defines the (Ct) threshold values corresponding to the different levels of viral load detected and, in many cases, test results are only given as qualitative positive/negative classification. When quantitative evaluation is performed, results are provided in several discrete, broad levels (e.g., high, medium, and low) equivalent to given ranges of viral concentrations. Notably, Ct values cannot be compared from one test to another or even within lots of the same test, and there is no consensus on the Ct values corresponding to disease severity or about any threshold to define the risk of contagion associated to an infected individual^80^.

### Hyperspectral imaging

An A-Series VNIR hyperspectral camera (Headwall, Massachusetts, USA) mounted with a 35-mm LM35HC lens (KOWA, Saitama, Japan) was used to obtain the reflectance spectra of the samples under study, as described in a previous work^37^. The spectral wavelengths ranged from 406.62 to 996.46 nm accounting for a total of 810 bands. The camera was placed on a motorized linear translation stage (Zaber, Vancouver, Canada) 30 cm above the sample plane which was illuminated using two ASD Illuminator halogen light sources (Malvern Panalytical, Worcestershire, UK), as illustrated in Fig. 2a. The light sources were positioned 35 cm over the sample plane with their irradiance axis in a 30-degree angle with the vertical line. To ensure the sample was homogenously illuminated, an area of 9 × 8 cm^2^ was scanned with a resolution of 1275 pixels by 1000 pixels. The hyperspectral images thus obtained were codified using 8 bits. For calibration, the white reference was a 3.62-inch Spectralon white reference (Labsphere, New Hampshire, USA), and the dark reference was obtained by blocking the imaging system with the cover provided by the manufacturer.

### Sample imaging

Hyperspectral images were obtained at room temperate with relative humidity between 40 and 60%, as described previously^37^. Concisely, 5-µL droplets were placed on a 22 mm × 22 mm polytetrafluoroethylene (PTFE) supporting plate (BSH, Seville, Spain) with a 1-mm thickness. After depositing the droplets acquire the approximately half-ellipsoidal profile given by the equilibrium of their weight, the supporting plate reaction, and the air-fluid surface tension. Assuming a simplified disc model of a droplet deposited on the supporting plate, each pixel of the droplet image corresponded^37^ to an equivalent volume of approximately 4 nL. For analysis of human samples, six droplets per subject were deposited on each imaging plate. Note sufficient space was left between neighboring droplets (about 5 mm) to avoid information cross-talk while facilitating digital segmentation. The supporting plate was positioned afterwards within the imaging area onto a 10-mm thick wood board to record the reflectance spectra.

### Image pre-processing

Hyperspectral images were segmented and pre-processed off-line^37^. Segmentation was performed using Evince (Prediktera, Umeå, Sweden). A pseudo red–green–blue (RGB) subset of the hyperspectral cube was built to determine the droplet contour following a conservative approach. Next, a principal component analysis (PCA) was performed on all pixel spectra within a droplet to remove outliers and foreign elements (e.g., bright reflections) from further analysis. It is important to note that the process of droplet segmentation and removal of unwanted imaging artefacts gives (slightly) different results according to the specific features of the droplet, and, consequently, the corresponding numbers of valid pixels for analysis also differ (for each droplet). Optical diffuse reflectance spectra were then converted to pseudo-absorbance spectra and normalized using the standard normal variate (SNV) transform. A Savitzky-Golay smoothing filter (order 5, with a 20-point window) was applied, followed by a correction of the baseline.

### PLS-DA models and classification

PLS-DA models are built (using the corresponding training sets) with a number of latent variables selected following a standard cross-validation schema^62^ to retain a certain percentage of the data variance. These type of models generate an output value of classification for each sample by applying a linear regression with the calculated model variables. Three different types of PLS-DA models (namely, Models 1, 2 and 3) were constructed for data processing. Their number of latent variables was selected to retain over 90% or 95% of the training data variance and provide the best discrimination among samples of different classes. The total number of patients was randomly split in k-fold cross validations (method of venetian blind, 20 data splits, with 4 samples per blind (thickness)). The training set corresponded to about a 62% of the cases, and the test set to 38% of the cases.

Two PLS-DA models of the first type (PLS-DA Model 1) were built (using Python 3.8.5 with Numpy, SKlearn, Pandas and Seaborn libraries) from individual pixel spectra (i.e., their inputs are the 810 values of reflectance registered by the hyperspectral camera at each pixel) with two latent variables to retain at least 95% of data variance and a tenfold cross validation. PLS-DA Models 1 were built using all pixels from all samples. They were employed in Experiment 1 (with synthetic SARS-CoV-2 viral models) and in Experiment 3 (with fresh saliva samples) to reduce the dimensionality of the spectral data, and to explore the distribution of positive and negative pixels in two-dimensional scatter plots with all processed pixels.

The PLS-DA Model of the second type (PLS-DA Model 2) was also built from individual pixel spectra (i.e., with the 810 values of reflectance recorded for each pixel), with 12 latent variables to retain at least 95% of the data variance. Differently from the models of the first type, it was built and tested using samples from independent training and test patient sets. It generated an output variable between 0 (negative) and 1 (positive) for classification of each pixel of the sample test set. A threshold value was selected from inspection of the corresponding boxplots (of the output variable) and ROC curves to determine the assigned result of classification per-pixel. This analysis was performed using PLS Toolbox 8.6 (Eigenvector Research Inc, Washington, USA) under Matlab R2020b (The Mathworks Inc., Massachusetts, USA). PLS-DA Model 2 was employed in Experiment 2-A (with nasopharyngeal exudate samples) to generate a per-pixel classification, later integrated at droplet and patient level.

The PLS-DA Model of the third type (PLS-DA Model 3) was constructed from a different approach, using patient-averaged spectra (i.e., inputs were one spectrum per patient, each one having the 810 values corresponding to the wavelengths registered by the image sensor) from the same training sets of patients used for PLS-DA Model 2 and for the FFNN. It was built with 10 latent variables to retain at least 90% of the data variance, and it generated an output variable between − 1 (negative) and + 1 (positive) for each subject (patient) of the same test set used for the other two models. A threshold value of the output variable was selected from inspection of the corresponding boxplots and ROC curve to determine the assigned result of classification per-patient.

Model 3 was employed in Experiment 2-A (also with nasopharyngeal exudate samples) to directly generate a per-patient classification to be compared with those resulting from the integration to patient-level of the outputs of the PLS-DA Model 2 and of the FFNN model. This analysis with PLS-DA Model 3 was developed using The Unscrambler X 10.4 (CAMO, Norway).

### FFNN model and classification

In Experiment 2-B, an additional approach was also employed: following the same procedure as in the previous study^37^, pixel spectra were processed to extract a set of 28 spectral feature descriptors in the following four spectral fringes of interest: 625–900 nm, 600–700 nm, 700–800 nm, 800–900 nm. These descriptors quantify morphological characteristics of the spectral curves in selected bands—also considering the spectrum of the background—and fed the inputs of a FFNN. Different configurations were tested to balance network complexity (number of neurons and hidden layers) and the available samples for training while achieving a high prediction accuracy. The FFNN was implemented in Matlab R2020b (The Mathworks Inc., Massachusetts, USA) with 20 hidden layers and one output layer^37^. The artificial neurons were implemented using sigmoid and softmax functions for hidden and output neurons respectively. Each hidden layer contained a number of neurons given by the product of the number of spectral feature descriptors and the number of fringes of interest in which they were obtained^37^ (typically, 28 × 4 = 112 neurons/layer).

The FFNN generated an output variable between 0 (negative) and 1 (positive) for each individual pixel. From the analysis of the corresponding boxplots and ROC curves of the training and validation sets, a per-pixel threshold was defined for classification. As in the previous study^37^, a supervised training was carried out using the scaled conjugate gradient backpropagation to find the optimal weights, limiting the number of iterations to 1000. The network performance was tested by evaluating the mean square error, and its values were plotted as a function of the number of training epochs (see Supplementary Information). To prevent overfitting, the best performance was identified as corresponding to the epoch with the lowest validation error.

The training set corresponded to 46% of cases, the validation set to 16% of the cases, and the test set to 38% of cases (on a per-patient basis). As mentioned, training and validation sets for the FFNN corresponded to the training set for the PLS-DA Models. The total number of subjects was randomly split in k-fold cross validations, and the same patient sets were employed for all analysis of Experiments 2-A and 2-B.

### Spectral feature descriptor (SFD)

Given the spectral pseudo-absorbance function of each pixel of the sample under analysis (y) and that of its supporting plate -defined as a reference- (y_ref_) in a certain wavelength interval, the spectral feature descriptor (SFD) was defined as a relative difference between both spectral functions in that interval as follows:

$$SFD=\frac{<{y- y}_{ref}>}{<y>+<{y}_{ref}>}.$$

This parameter was calculated in the following spectral fringes: For samples with lentiviral particles in 483–610 nm, for exudates in 870–910 nm, and for samples of fresh saliva in 483–610 nm and 407–470 nm.

### Data analyses

Random splits (here identified as Trials) for assignment of sets and for the k-fold cross-validation were performed on a per-droplet basis (in Experiment 1) and in a per-patient basis (in Experiments 2 and 3) to avoid that pixels from the same droplet (in Experiment 1) or from the same patient (in Experiments 2 and 3) were assigned to different sets. In addition, the same patient sets were used for Experiments 2-A and 2-B.

Confusion matrices and the corresponding values of sensitivity and specificity and ROC curves were obtained to assess the goodness of classification at three levels: pixel, droplet, and subject. The accuracy of the prediction was assessed using the area under the ROC curve (AUROC) value. Classification thresholds were obtained from ROC curves as the values that minimized the quadratic distance to the point with highest sensitivity (true positive rate) and specificity (true negative rate). The use of confusion matrices and AUROC values (instead of other statistical features) for the evaluation of PLS-DA models improves their performance for detecting small differences^62^.

Per-pixel classification results (from PLS-DA Model 2 and from the FFNN) were integrated per-droplet and per-patient. This was accomplished by defining two additional thresholds which defined, respectively, the percentage of pixels (of a given droplet) and of droplets (of a given patient) required to be positive to classify the corresponding droplet or patient as positive.

Normality was tested using the Kolmogorov–Smirnoff test at 95% significance. Wilcoxon rank sum tests were used for comparison of non-normal distributions. To characterize statistical distributions, we used box plots displaying the median values and the inter-quartile ranges. The whiskers were used to show 2.7 times the standard deviation, and the outliers beyond that range were removed for clarity. Here, p-values below 0.0001 were considered as zero.

## Supplementary Information

Supplementary Information.

## Data Availability

All data needed to evaluate the findings are present in the paper and the Supplementary Materials. Additional data related to this work are available from the authors under reasonable request.
