# Supplementary material for: Optical imaging spectroscopy for rapid, primary screening of SARS-CoV-2: a proof of concept
Source: Sci Rep. 2022 Feb 18;12:2356. doi: 10.1038/s41598-022-06393-3 (PMC8857323; doi:10.1038/s41598-022-06393-3)
Supplement: Supplementary file 1 — Supplementary Information. [file 41598_2022_6393_MOESM1_ESM.docx]

SUPPLEMENTARY INFORMATION FOR

Article: “Optical imaging spectroscopy for rapid primary screening of SARS-CoV-2: a proof of concept”.

Authors:

Emilio Gomez-Gonzalez*^1,2^, Alejandro Barriga-Rivera^1,3^, Beatriz Fernandez-Muñoz^4^, Jose Manuel Navas-Garcia^5^, Isabel Fernandez-Lizaranzu^1,2^, Francisco Javier Munoz-Gonzalez^1^, Ruben Parrilla-Giraldez^6^, Desiree Requena-Lancharro^1^, Pedro Gil-Gamboa^1^, Cristina Rosell-Valle^2,4^, Carmen Gomez-Gonzalez^7,8^, Maria Jose Mayorga-Buiza^2,9,10^, Maria Martin-Lopez^2,4^, Olga Muñoz^11^, Juan Carlos Gomez-Martin^11^, Maria Isabel Relimpio-Lopez^10,12,13^, Jesus Aceituno-Castro^11,14^, Manuel A. Perales-Esteve^15^, Antonio Puppo-Moreno^7,8^, Francisco Jose Garcia-Cozar^16^, Lucia Olvera-Collantes^17^, Raquel Gomez-Diaz^2^, Silvia de los Santos-Trigo^18^, Monserrat Huguet-Carrasco^19^, Manuel Rey^20^, Emilia Gomez^21^, Rosario Sanchez-Pernaute^4^, Javier Padillo-Ruiz^2,10,22^, Javier Marquez-Rivas^2,10,23,24^.

1. Department of Applied Physics III, ETSI School of Engineering, Universidad de Sevilla; 41092 Sevilla, Spain.
2. Institute of Biomedicine of Seville (IBIS); 41013 Sevilla, Spain.
3. School of Biomedical Engineering, The University of Sydney; NSW 2006, Australia.
4. Unidad de Producción y Reprogramación Celular (UPRC), Red Andaluza de Diseño y Traslación de Terapias Avanzadas, Consejería de Salud y Familias, Junta de Andalucía; 41092 Sevilla. Spain.
5. EOD-CBRN Group, Spanish National Police; 41011 Sevilla, Spain.
6. Technology and Innovation Centre, Universidad de Sevilla; 41012 Sevilla, Spain.
7. Service of Intensive Care, University Hospital ‘Virgen del Rocio’; 41013 Sevilla, Spain.
8. Department of Medicine, College of Medicine, Universidad de Sevilla; 41009 Seville, Spain.
9. Service of Anesthesiology, University Hospital ‘Virgen del Rocio’; 41013 Sevilla, Spain.
10. Department of Surgery, College of Medicine, Universidad de Sevilla; 41009 Seville, Spain.
11. Instituto de Astrofísica de Andalucía, CSIC; 18008 Granada, Spain.
12. Department of Ophthalmology, University Hospital ‘Virgen Macarena’; 41009 Sevilla, Spain.
13. OftaRed, Institute of Health ‘Carlos III’; 28029 Madrid, Spain.
14. Centro Astronomico Hispano Alemán; 04550 Almeria, Spain.
15. Department of Electronic Engineering, ETSI School of Engineering, Universidad de Sevilla; 41092 Sevilla, Spain.
16. Department of Biomedicine, Biotechnology and Public Health, University of Cadiz; 11003 Cadiz, Spain.
17. Instituto de Investigación e Innovación Biomedica de Cádiz (INIBICA); 11009 Cadiz, Spain.
18. Corporación Tecnológica de Andalucía; 41092 Sevilla, Spain.
19. CER ‘Dr. Gregorio Medina Blanco’; 41807 Espartinas, Sevilla, Spain.
20. CAMBRICO BIOTECH; 41015 Sevilla, Spain.
21. Joint Research Centre, European Commission; 41092 Sevilla, Spain.
22. Department of General Surgery, University Hospital ‘Virgen del Rocío’; 41013 Sevilla, Spain.
23. Service of Neurosurgery, University Hospital ‘Virgen del Rocío’; 41013 Sevilla, Spain.
24. Centre for Advanced Neurology; 41013 Sevilla, Spain.

Correspondence:

Prof. Emilio Gomez-Gonzalez

Department of Applied Physics III

School of Engineering, Universidad de Sevilla

Camino de los Descubrimientos s/n, 41092 Sevilla, Spain,

[egomez@us.es](mailto:egomez@us.es)

***Supplementary Material and Methods***

The three independent experiments were performed following the flow charts detailed in Figures 1s, 2s, 3s, and 4s.

Experiment 1 (Figure 1s) presents an analysis of two synthetic models of SARS-CoV-2 (lentiviral particles expressing the characteristic spike protein of the SARS-CoV-2 and the G glycoprotein of the vesicular stomatitis virus) and their negative controls, in four levels of concentration (viral load), in two biofluids (phosphate buffered saline solution and artificial saliva). Experiment 2 (Figures 2s and 3s) presents the analysis of inactivated nasopharyngeal exudate samples of SARS-CoV-2-positive *symptomatic* patients (in three viral loads) and their negative *symptomatic* controls; and Experiment 3 (Figure 4s) presents the analysis of fresh saliva samples of SARS-CoV-2-positive *asymptomatic* patients and negative *asymptomatic* controls.

Their corresponding tests are carried out as follows: in Experiment 1, on synthetic viral models, the analysis is performed using a per-pixel PLS-DA model and the calculation of a spectral feature descriptor. In Experiment 2, on inactivated nasopharyngeal exudate samples, the classification analysis is performed using i) a per-pixel PLS-DA model, integrated to per-droplet and per-patient levels, ii) a per-pixel FFNN model, also integrated to per-droplet and per-patient levels, iii) a per-patient PLS-DA model built on patient-averaged spectra (the three of them tested on the same patient sets) and iv) a per-pixel calculation of the spectral feature descriptor. Finally, in Experiment 3, on fresh saliva samples, the analysis is again performed using a per-pixel PLS-DA model and the calculation of a spectral feature descriptor.

| 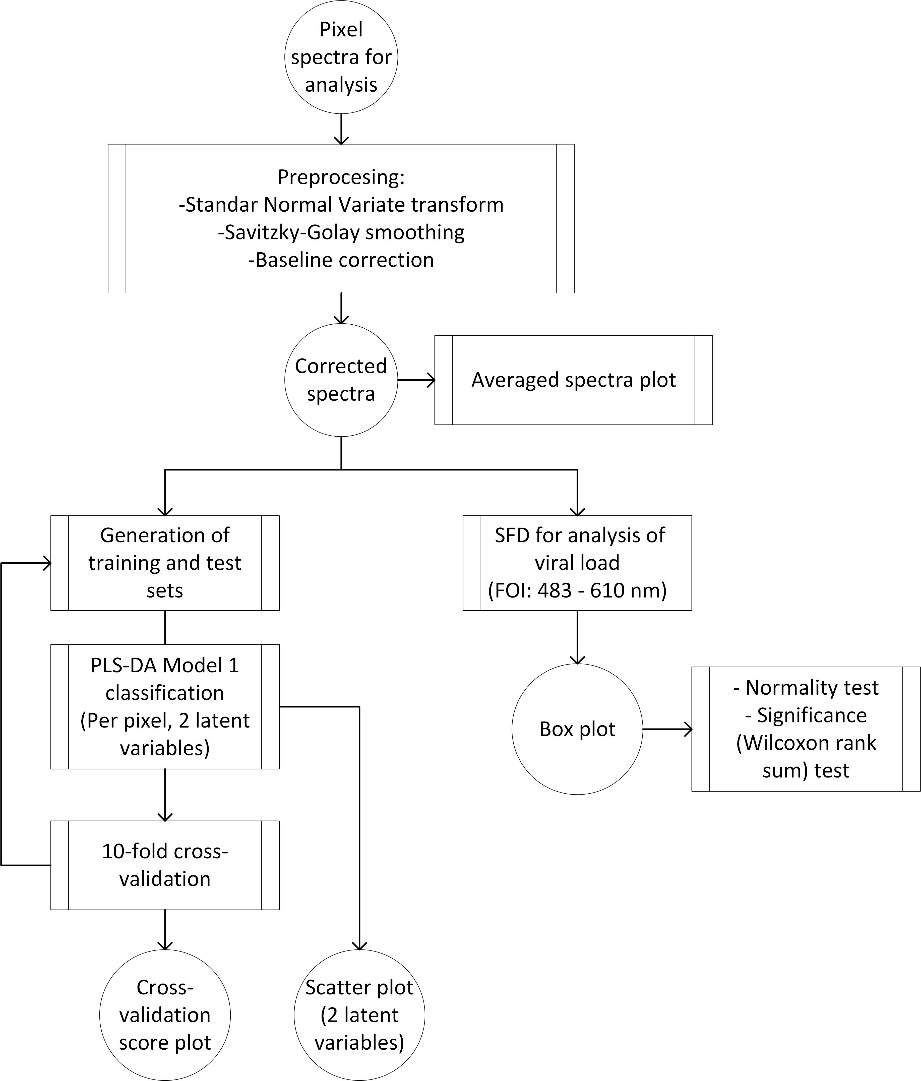 |
| --- |
| **Figure 1s. Flow chart of Experiment 1: Analysis of two synthetic viral models and their negative controls, in four levels of concentration (viral load), in two biofluids (phosphate buffered solution and artificial saliva).** Per-pixel classification is given by PLS-DA Model 1 (two latent variables, 10-fold cross-validation). Viral load is analyzed by calculating SFD in selected FOI. Averaged spectra plot, 2-latent variables scatter plot of PLS-DA Model 1 and SFD box plots (with statistical normality and significance tests) are generated. PLS-DA = partial least square-discriminant analysis. S-LP = lentiviral particles pseudotyped with the SARS-CoV-2 Spike protein. G-LP = lentiviral particles pseudotyped with the vesicular stomatitis virus G protein. SFD = spectral feature descriptor. FOI = (spectral) fringe of interest. |

| 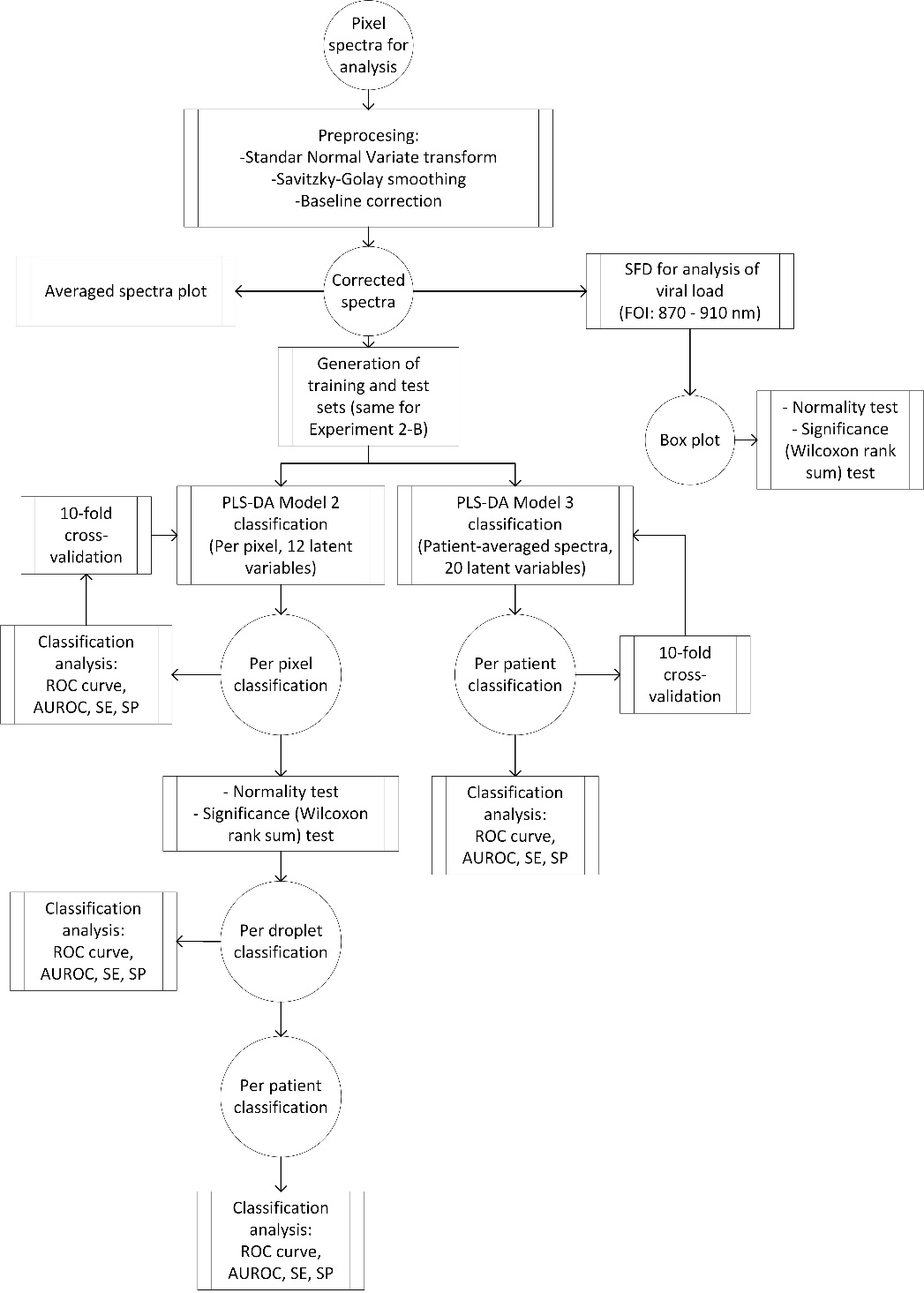 |
| --- |
| **Figure 2s. Flow chart of Experiment 2-A: Analysis of nasopharyngeal exudates of SARS-CoV-2 qRT-PCR positive *symptomatic* patients (in three viral loads) and negative *symptomatic* controls using two different PLS-DA models.** A per-pixel classification is given by PLS-DA Model 2 (12 latent variables), later integrated to per-droplet and per-patient levels, and an independent PLS-DA Model 3 (20 latent variables) is constructed from patient-averaged spectra to produce a directly per-patient classification. Viral load is analyzed by calculating SFD in selected FOI. Averaged spectra plots and SFD box plots (with statistical normality and significance tests) are generated. PLS-DA = partial least square-discriminant analysis. SFD = spectral feature descriptor. FOI = (spectral) fringe of interest. qRT-PCR = quantitative reverse transcription polymerase chain reaction (PCR). ROC = receiver operating characteristic (curve). AUROC = area under the ROC curve. SE = sensitivity. SP = specificity. Patient sample sets are the same of Experiment 2-B. |

| 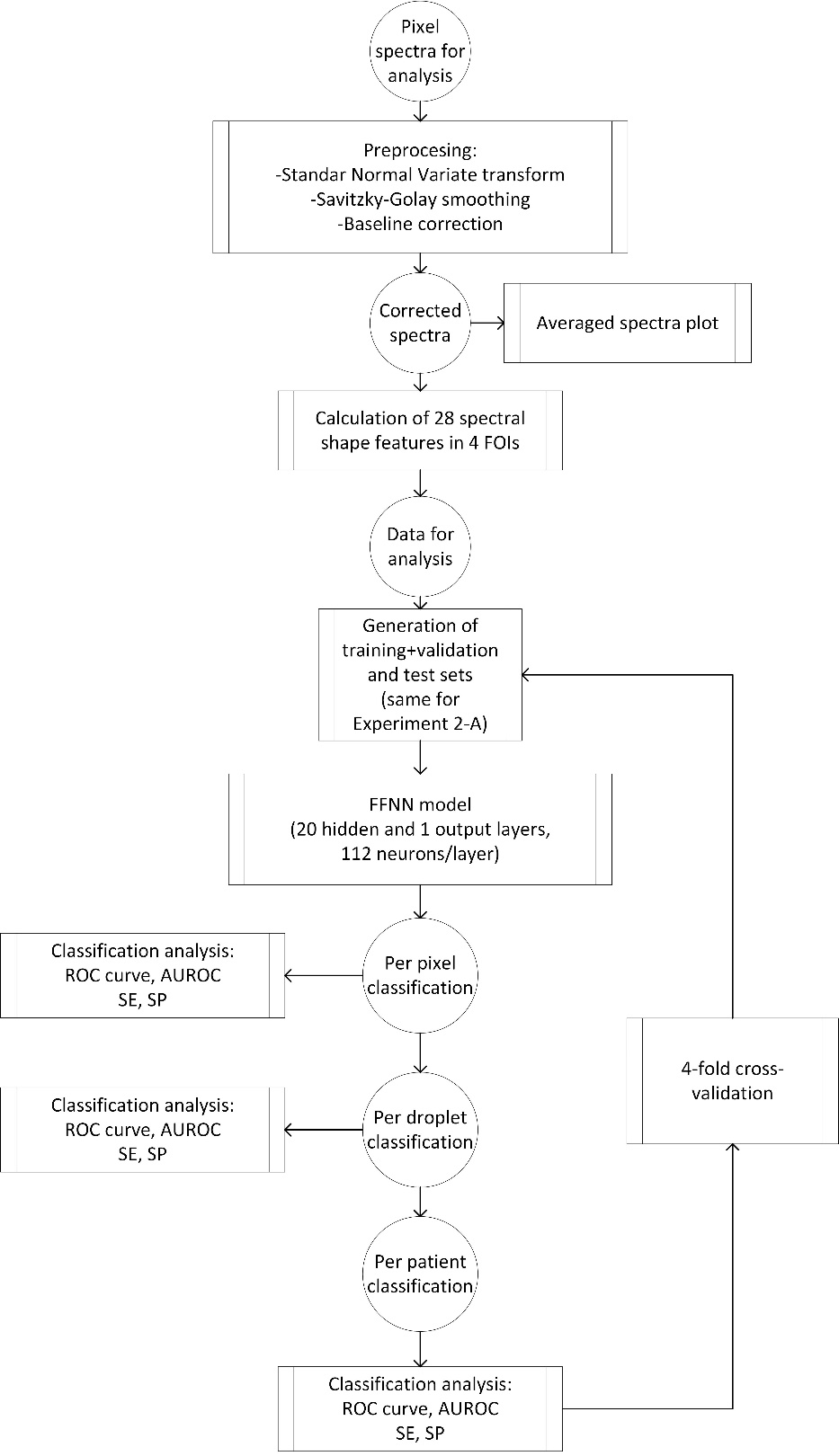 |
| --- |
| **Figure 3s. Flow chart of Experiment 2-B: Analysis of nasopharyngeal exudate samples of SARS-CoV-2 qRT-PCR positive *symptomatic* patients (in three viral loads) and negative *symptomatic* controls.** Per-pixel classification is given by a feed-forward neural network (4-fold cross validation), later integrated to per-droplet and per-patient levels. FFNN = feed-forward neural network. SFD = spectral feature descriptor. FOIs = (spectral) fringes of interest. qRT-PCR = quantitative reverse transcription polymerase chain reaction (PCR). ROC = receiver operating characteristic (curve). AUROC = area under the ROC curve. SE = sensitivity. SP = specificity. Patient sample sets are the same of Experiment 2-A. |

| 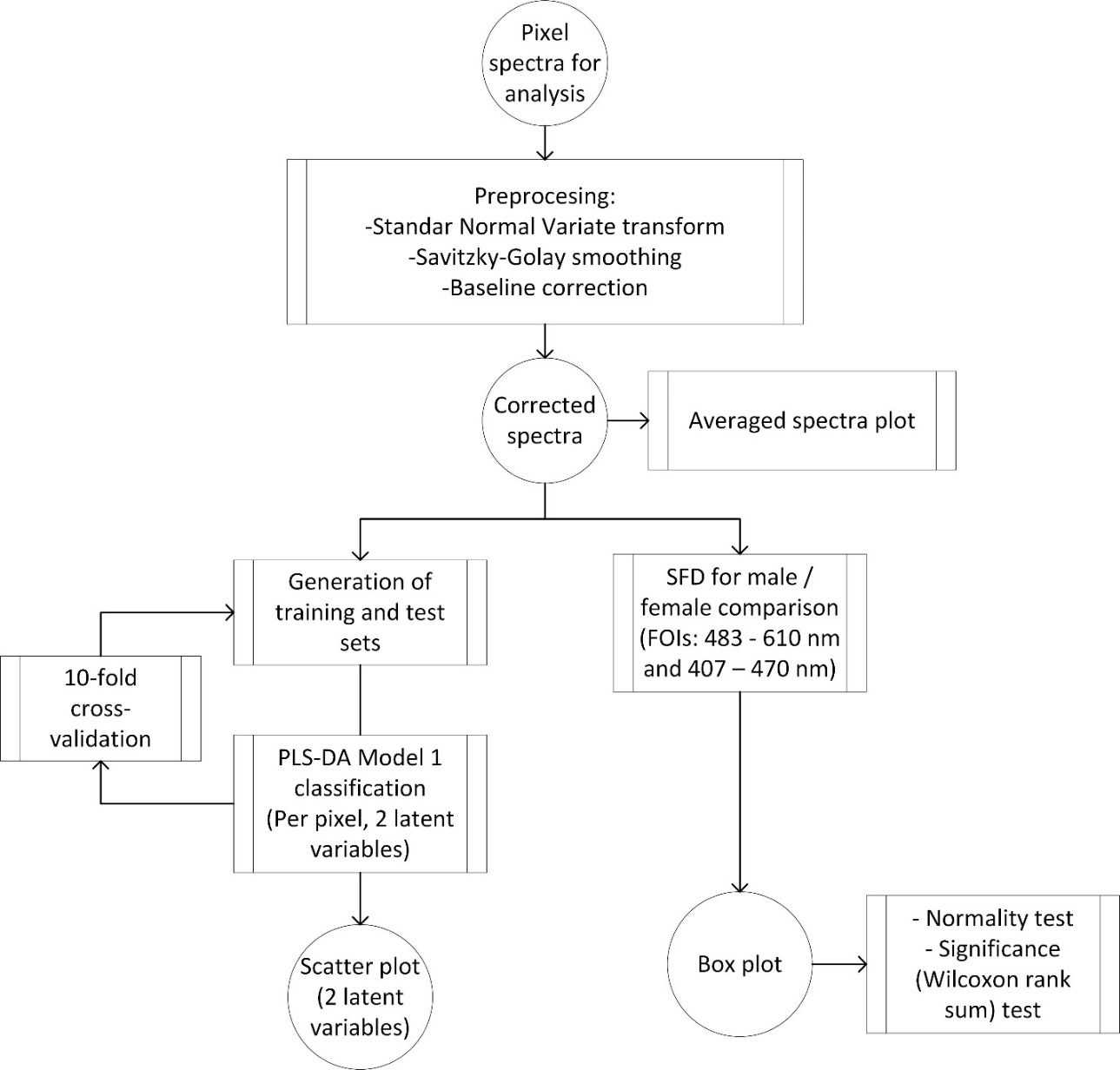 |
| --- |
| **Figure 4s. Flow chart of Experiment 3: Analysis of fresh saliva samples of SARS-CoV-2 PCR-positive asymptomatic patients and PCR-negative asymptomatic controls.** Per-pixel classification is given by PLS-DA Model 1 (two latent variables, 10-fold cross-validation). Male-female comparison is performed by calculating SFD in selected FOIs. Averaged spectra plot, 2-latent variables scatter plot of PLS-DA Model 1 and SFD box plots (with statistical normality and significance tests) are generated. PLS-DA = partial least square-discriminant analysis. SFD = spectral feature descriptor. FOIs = (spectral) fringes of interest. PCR = polymerase chain reaction test. |

***Supplementary Results***

Experiment 2-A: PLS-DA model using patient-averaged spectra (PLS-DA Model 3)

The PLS-DA Model 3 of Experiment 2-A was built upon the averaged pixel spectra per patient. It was constructed and tested using the same training and test groups of the Trial 1 used for the per-pixel classification models (PLS-DA Model 2 and FFNN) described in the manuscript.

Figure 5s shows the receiver operating characteristic curve of the per-patient classification given by PLS-DA Model 3 for Trial 1 (threshold = - 0.47). Table Is shows the confusion matrix, and Figure 6s the values of the output variable for each subject of the test set. Samples with uncertainties in their values which include zero have been assigned to (positive or negative) classes according to their classification outputs. Figure 7s shows the plot of explained variance for the training and test sets.

| 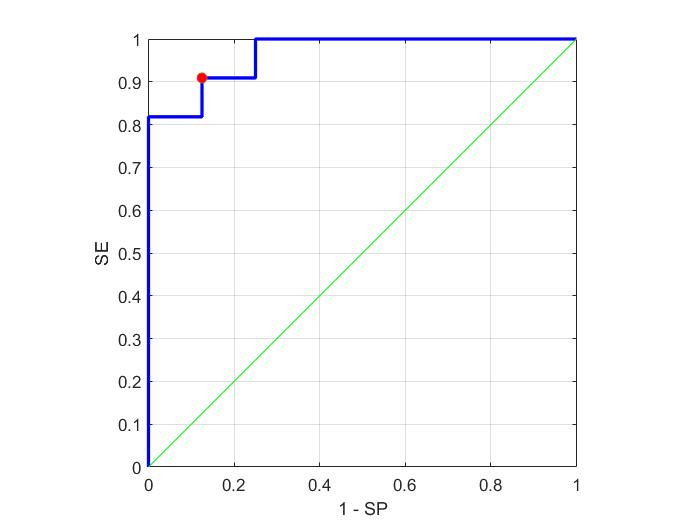 |
| --- |
| **Figure 5s. Receiver operating characteristic (ROC) curve of the per-patient classification of the test set of Trial 1 given by PLS-DA Model 3.** Red dot corresponds to a threshold value = -0.47. SE = 90.9%, SP = 87.5% and AUROC = 0.97. |

|  | | PCR test | | TOTAL |
| --- | --- | --- | --- | --- |
|  |  | Positive | Negative |  |
| PLS-DA classification | Positive | 10 | 1 | 11 |
|  | Negative | 2 | 14 | 16 |
|  | TOTAL | 12 | 15 | 27 |
| **Table Is. Confusion matrix corresponding to the results given by the PLS-DA Model 3 built upon per-patient averaged spectra for the test set of Trial 1.** | | | | |

| 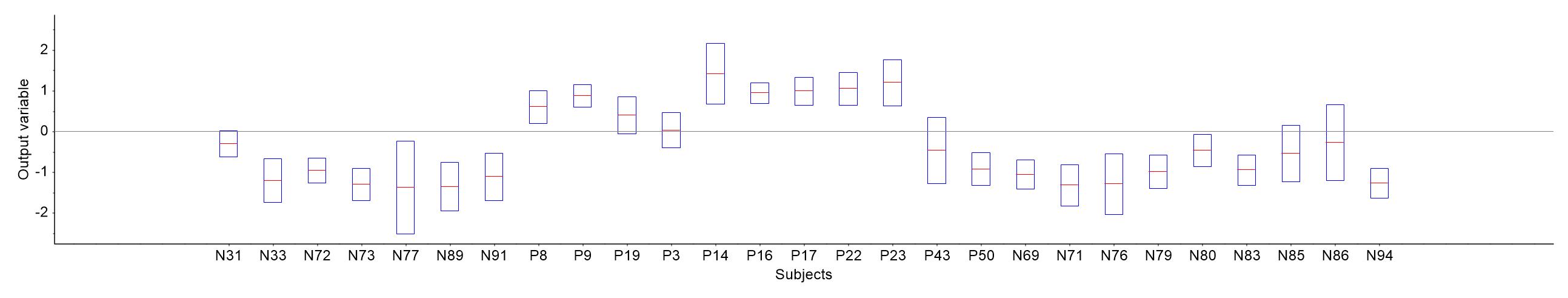 |
| --- |
| **Figure 6s. Classification results per patient obtained by PLS-DA Model 3 (Trial 1).** The values of the output variable for per-patient are shown. Boxplots represent the predicted values and the estimated uncertainties for each positive (P) and negative (N) subject of the test set. |

| **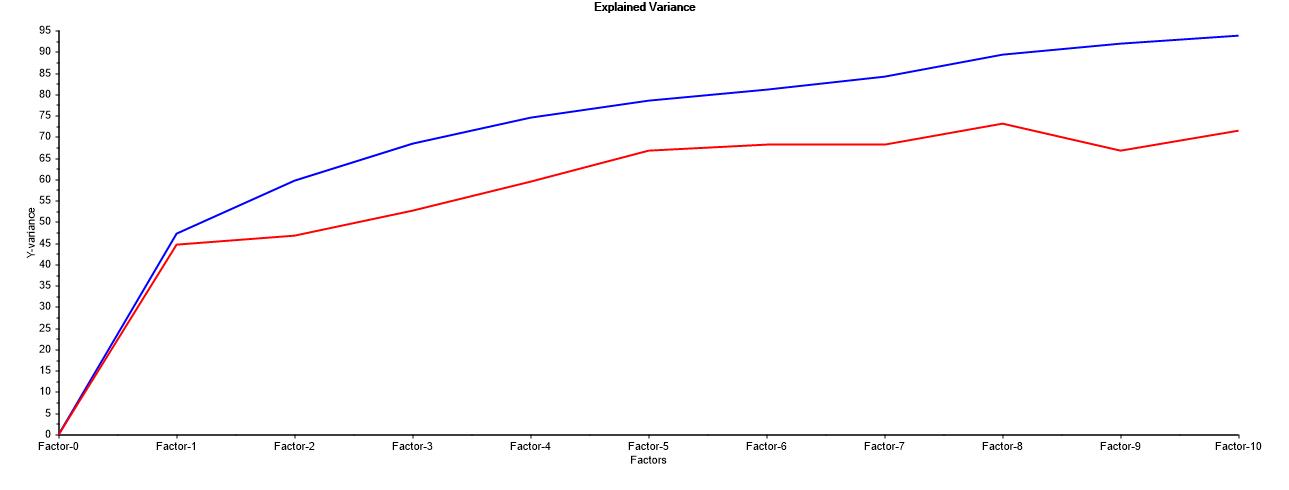** |
| --- |
| **Figure 7s. Explained variance plot of the PLS-DA Model 3 in Experiment 2 (Trial 1).** Blue line corresponds to the training set and red line to the test set. Using the 10 factors of the model, they are 93.8% for the training set and 71.46% for the test set. |

Experiment 2-B: FFNN cross-validation in exudate samples

Four different trials were developed to verify the results obtained by the feed forward neural network (FFNN) in Experiment 2-B. In addition to the subject grouping described in the manuscript (Trial 1), available samples were distributed in other three different configurations (Trials 2, 3 and 4). Training, validation, and test splits were randomly assigned for each one.

Table IIs shows the distribution of samples of Trials 2, 3 and 4, and Table IIIs the sensitivity, specificity, and area under the receiver operating characteristic curve obtained for each configuration (Trials 2, 3 and 4)

Figures 8s, 9s and 10s show the values of the output variable for per-pixel classification, and the corresponding results (receiver operating characteristic) at each level (pixel, droplet, and patient) for Trials 2, 3 and 4.

|  | | **PIXEL** | | | | **DROPLET** | | | | **PATIENT** | | | |
| --- | --- | --- | --- | --- | --- | --- | --- | --- | --- | --- | --- | --- | --- |
|  |  | **Pos** | | | **Neg** | **Pos** | | | **Neg** | **Pos** | | | **Neg** |
|  |  | **H** | **M** | **L** |  | **H** | **M** | **L** |  | **H** | **H** | **L** |  |
| **Trial 2** | **Tra** | 87962 | 30328 | 36708 | 174588 | 48 | 18 | 24 | 102 | 8 | 3 | 4 | 17 |
|  | **Val** | 25195 | 15994 | 8613 | 69424 | 12 | 12 | 6 | 42 | 2 | 2 | 1 | 7 |
|  | **Test** | 81533 | 22295 | 11803 | 167287 | 48 | 12 | 6 | 102 | 8 | 2 | 1 | 17 |
| **Trial 3** | **Tra** | 90791 | 18766 | 45321 | 172349 | 48 | 12 | 30 | 102 | 8 | 2 | 5 | 17 |
|  | **Val** | 29948 | 19523 | 0 | 74773 | 18 | 12 | 0 | 42 | 3 | 2 | 0 | 7 |
|  | **Test** | 73951 | 30328 | 11803 | 164177 | 42 | 18 | 6 | 102 | 7 | 3 | 1 | 17 |
| **Trial 4** | **Tra** | 95320 | 29718 | 36821 | 171178 | 48 | 18 | 24 | 96 | 8 | 3 | 4 | 16 |
|  | **Val** | 26963 | 8203 | 9047 | 76399 | 18 | 6 | 6 | 48 | 3 | 1 | 1 | 8 |
|  | **Test** | 72407 | 30696 | 11256 | 163722 | 42 | 18 | 6 | 102 | 7 | 3 | 1 | 17 |
| **Table IIs. Sample distribution of nasopharyngeal exudates at different levels (total numbers of patients, droplets and pixels) in the Training (Tra), Validation (Val), and Test groups for each configuration (Experiment 2-B, Trials 2, 3 and 4).** Positive (Pos) and negative (Neg) cases were determined by qRT-PCR. Positive cases include three viral load levels (high, H = 10^6^ copies·mL^-1^; medium, M = 10^4^ copies·mL^-1^ and low, L = 10^2^ copies·mL^-1^). | | | | | | | | | | | | | |

| **FFNN** | **Trial 2** | | | | **Trial 3** | | | | **Trial 4** | | | |
| --- | --- | --- | --- | --- | --- | --- | --- | --- | --- | --- | --- | --- |
|  | **Th** | **SE (%)** | **SP (%)** | **AUROC** | **Th** | **SE (%)** | **SP (%)** | **AUROC** | **Th** | **SE (%)** | **SP (%)** | **AUROC** |
| **Pixel** | 0.53 | 80.7 | 75.9 | 0.852 | 0.51 | 85.9 | 82.2 | 0.905 | 0.49 | 87.7 | 80.7 | 0.895 |
| **Droplet** | 0.65 | 98.5 | 83.3 | 0.925 | 0.69 | 98.5 | 89.3 | 0.962 | 0.85 | 95.5 | 88.2 | 0.939 |
| **Patient** | 0.90 | 90.9 | 94.1 | 0.936 | 0.80 | 100 | 94.1 | 0.941 | 0.80 | 90.9 | 94.1 | 0.984 |
| **Table IIIs. Sensitivity (SE), specificity (SP) and area under the receiving operating characteristic (AUROC) curve obtained for each additional experimental configuration (Trials 2, 3 and 4) of the nasopharyngeal exudate samples (Experiment 2-B, Trials 2, 3 and 4).** Note that classification algorithms provide results as (per-pixel, per-droplet and per-patient) ‘positive’ (with any level of viral load) or ‘negative’ assignations. Threshold (Th) values are shown for per-pixel, per-droplet and per-patient classification. | | | | | | | | | | | | |

| **Trial 2** |
| --- |
| 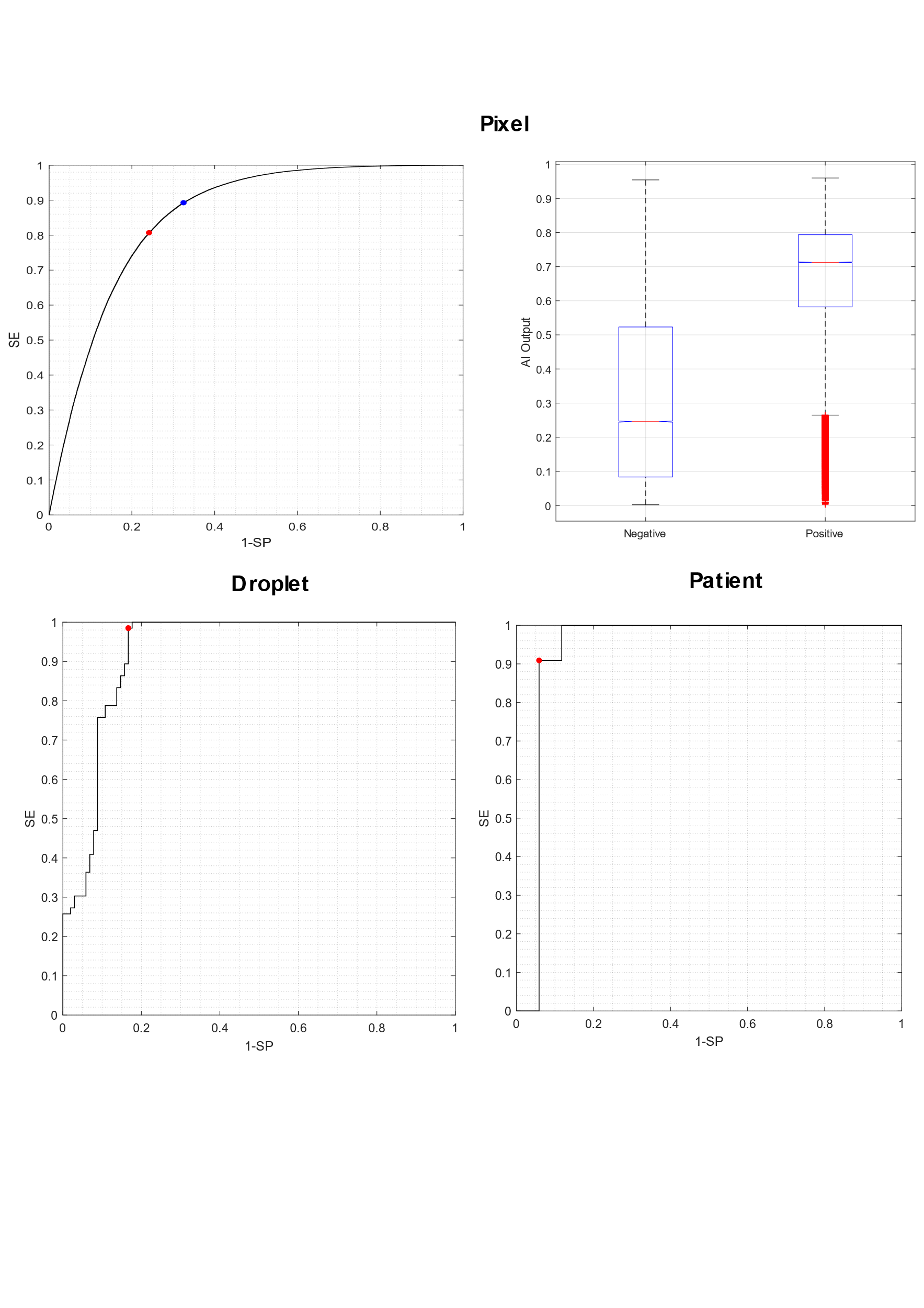 |
| **Figure 8s. Results for the additional configuration (Experiment 2-B, Trial 2) of the nasopharyngeal exudate samples processed using the FFNN methodology.** Receiving operating characteristic curves are shown at pixel, droplet and patient levels. Red dots correspond to optimal values of sensitivity (SE) and specificity (SP). The values of the output variable for per-pixel classification are shown (boxplots) for positive and negative samples. Red crosses represent the outliers. |

| **Trial 3** |
| --- |
| 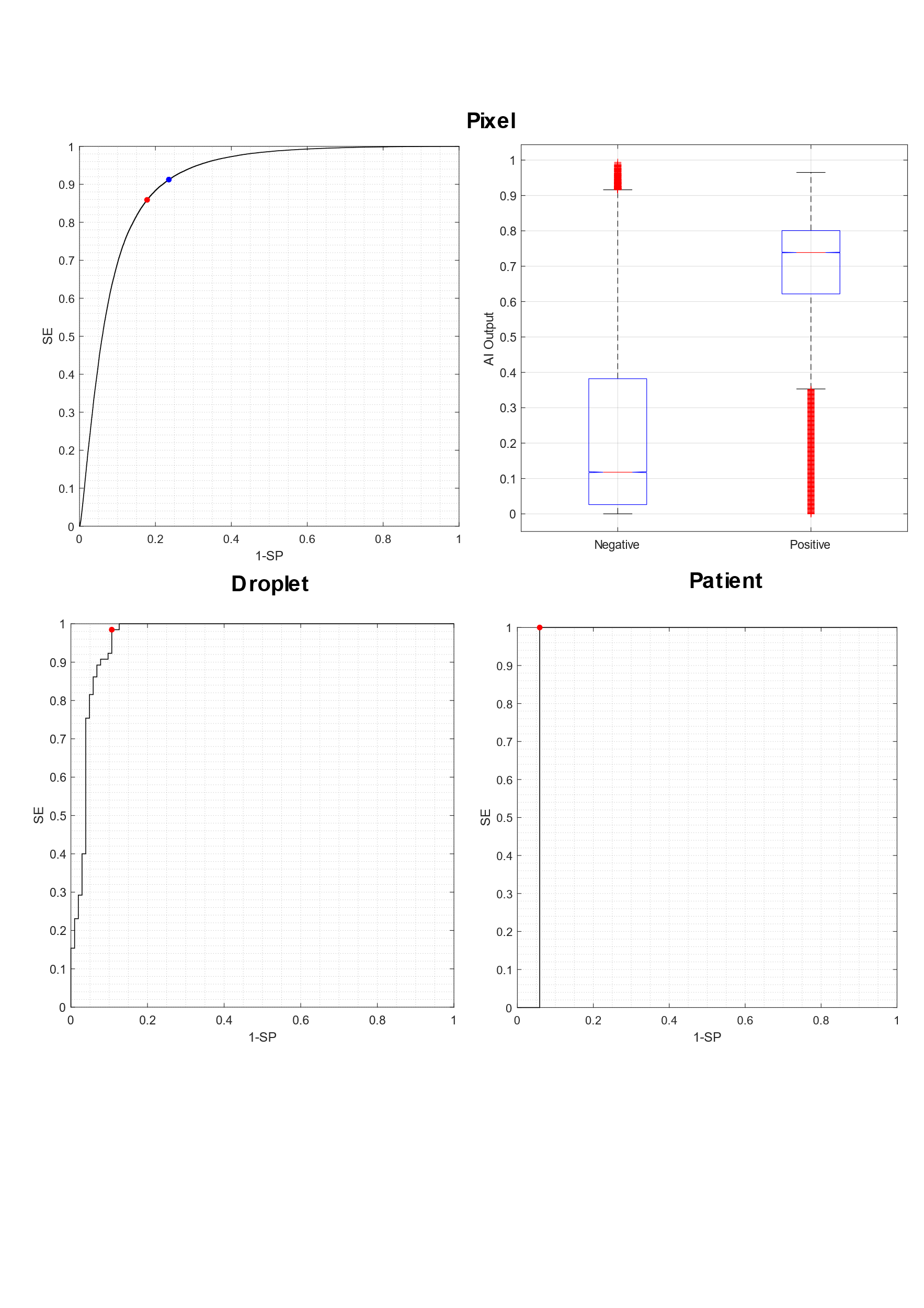 |
| **Figure 9s. Results for the additional configuration (Experiment 2-B, Trial 3) of the nasopharyngeal exudate samples processed using the FFNN methodology.** Receiving operating characteristic curves are shown at pixel, droplet and patient levels. Red dots correspond to optimal values of sensitivity (SE) and specificity (SP). The values of the output variable for per-pixel classification are shown (boxplots) for positive and negative samples. Red crosses represent the outliers. |

| **Trial 4** |
| --- |
| **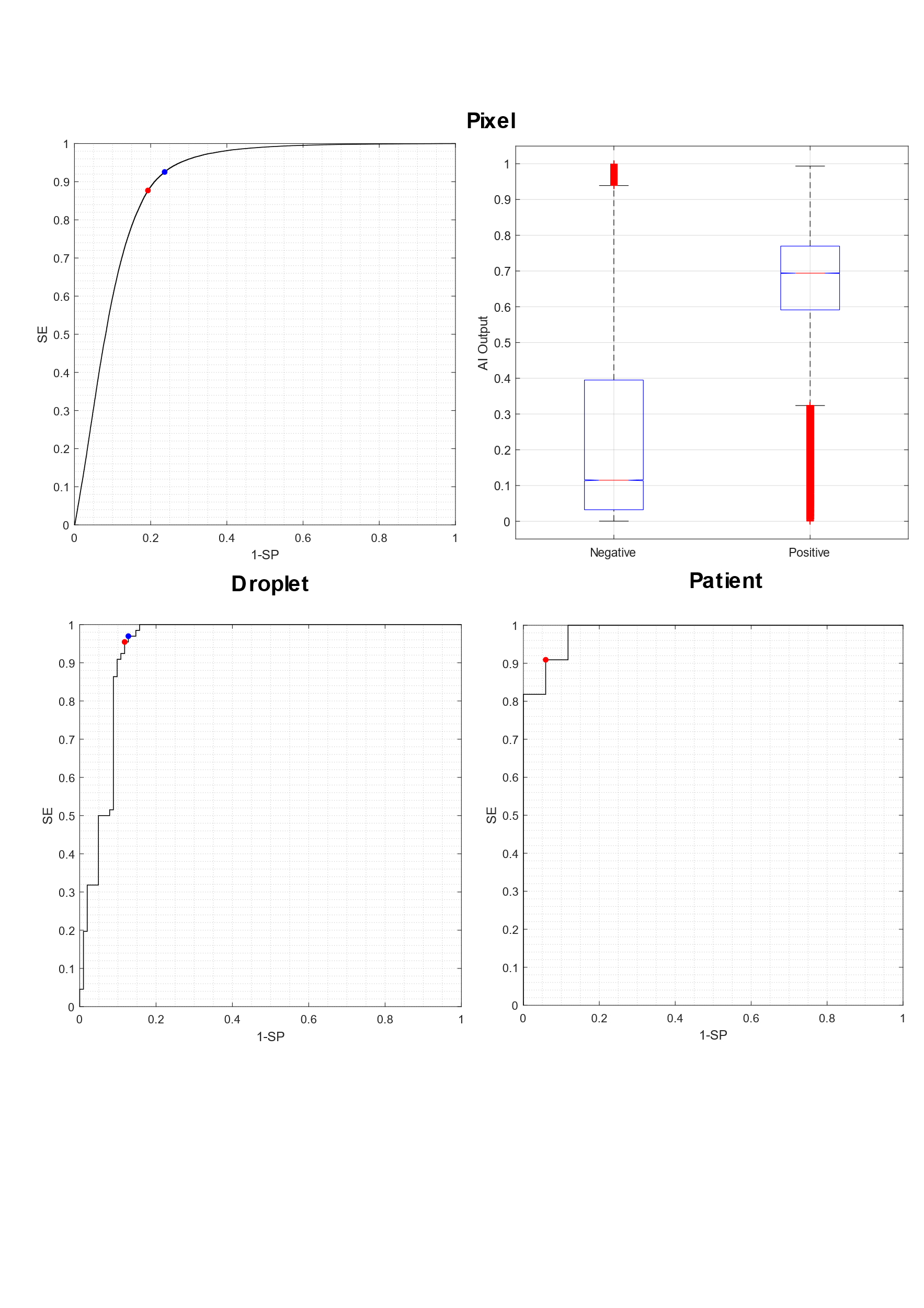** |
| **Figure 10s. Results for the additional configuration (Experiment 2-B, Trial 4) of the nasopharyngeal exudate samples processed using the FFNN methodology.** Receiving operating characteristic curves are shown at pixel, droplet, and patient levels. Red dots correspond to optimal values of sensitivity (SE) and specificity (SP). The values of the output variable for per-pixel classification are shown (boxplots) for positive and negative samples. Red crosses represent the outliers. |

Experiment 2-B: FFNN overfit tests for classification of exudate samples

Figure 11s shows the network performance as a function of the number of training epochs (see Supplementary Information). To prevent overfitting, the best validation performance was identified as corresponding to the epoch 96, with the lowest error.

| 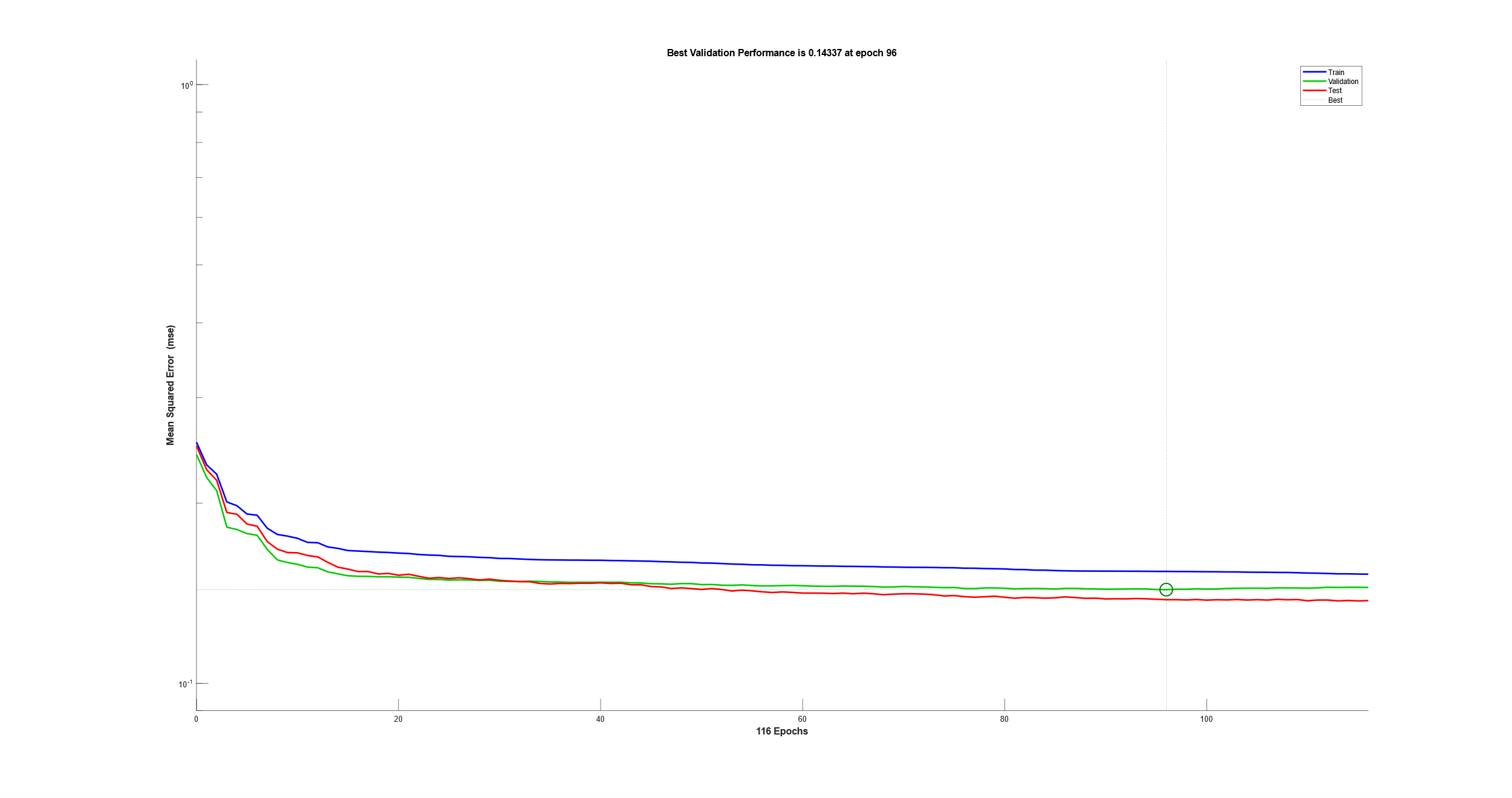 |
| --- |
| **Figure 11s. Overfit tests for classification of exudate samples (Experiment 2-B, Trial 1).** Mean squared error for training (blue line) validation (green line) and test (red line) calculated in each epoch.116 epochs were obtained and the best result was in epoch 96. |
